# Targeting STAU1 prevents p53 apoptotic signaling in neurodegeneration

**DOI:** 10.1038/s41419-025-08067-0

**Published:** 2025-10-27

**Authors:** Mandi Gandelman, Sharan Paul, Karla P. Figueroa, Justine Sundrud, Warunee Dansithong, Daniel R. Scoles, Stefan M. Pulst

**Affiliations:** Department of Neurology, University of Utah, Salt Lake City, UT USA

**Keywords:** Amyotrophic lateral sclerosis, Stress signalling

## Abstract

Stress responses and neuronal death mediated by the p53 pathway play a central role in the progression of neurodegenerative disease, constituting a common target to extend neuronal function and survival. Interaction of p53 and its signaling network with RNA-binding proteins (RBPs) helps fine-tune its activation and the resulting cell fates. Preclinical therapeutics based on depletion of the RBP STAUFEN-1 (STAU1) protein successfully prevent neurodegeneration, however, the specific mechanisms are not fully understood. STAU1 is pathologically overabundant in multiple neurological disorders and contributes to neurodegeneration by exacerbating autophagy dysfunction, endoplasmic reticulum stress, and RNA-protein condensate accumulation. We previously showed that lowering STAU1 levels mitigates these disease-related features and prevents neuronal death in animal models of amyotrophic lateral sclerosis/frontotemporal dementia (ALS/FTD) and spinocerebellar ataxia type 2 (SCA2). Here, we show by combined transcriptomic and functional analyses that STAU1 reduction results in the inhibition of apoptosis through the p53 pathway. In both proliferating and post-mitotic cell types—human iPSC-derived neurons, mouse cortical neurons, SH-SY5Y cells, and fibroblasts—STAU1 reduction effectively prevented p53-mediated apoptosis and DNA damage induced by Nutlin-3 and etoposide. Further examination in C9orf72-expanded patient-derived fibroblasts and a C9orf72 mouse model of ALS/FTD, which exhibit baseline overabundance of STAU1 and activation of the p53 pathway, confirmed that STAU1 reduction also prevented p53-driven pro-apoptotic signaling. These findings establish STAU1 as a novel modulator of DNA damage and p53-dependent apoptosis, suggesting that targeting STAU1 could be a promising approach to prevent neurodegeneration in ALS/FTD.

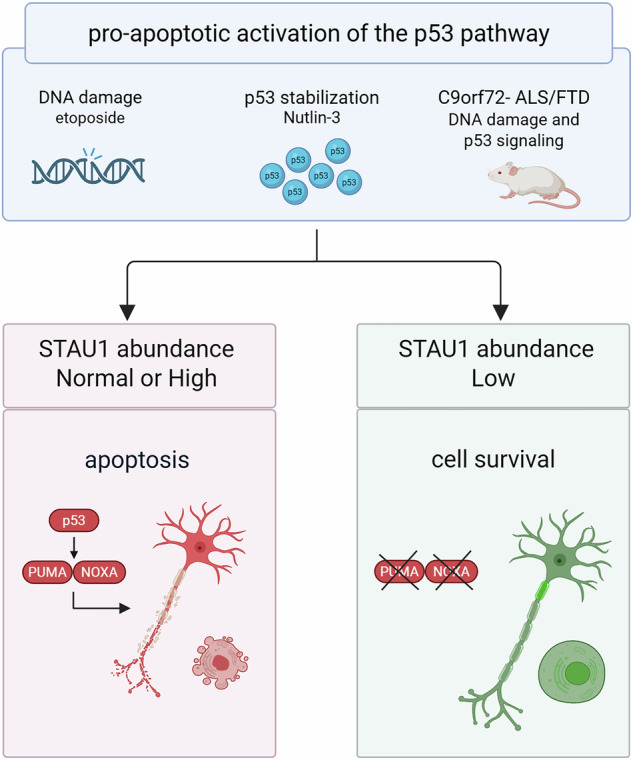

## Background

The p53 pathway is a major signaling hub that determines cell fates in response to stress, and chronic activation of p53 in neurons can lead to neuronal dysfunction and death [1, 2]. Increasing evidence of p53-mediated apoptosis in ALS/FTD make it a valuable therapeutic target to reduce maladaptive stress responses and delay neuronal death [1–3]. Repeat expansions in the C9orf72 gene cause ALS/FTD, associated with DNA damage and p53-mediated neuronal death, and ablation of p53 prevents neuronal death and extends the lifespan of multiple animal models [4–9].

Interaction of p53 and its signaling network with RBPs help fine-tune the activation of the p53 pathway and the resulting responses and cell fates. RBPs interact with the p53 mRNA and regulate its stability, translation, splicing and interactions with non-coding RNAs, and with the p53 protein, regulating its localization and degradation [10–12]. STAUFEN1 (STAU1) is a double-stranded RNA binding protein (RBP) with functions in RNA translation, splicing, stability, localization and decay [13–15]. STAU1 becomes highly overabundant in multiple human neurological diseases, including amyotrophic lateral sclerosis (ALS), frontotemporal dementia (FTD), Parkinson’s disease, Alzheimer’s disease and spinocerebellar ataxia type 2 (SCA2) [14, 16–24]; and also increases in response to acute stress [17–20, 22, 23]. STAU1 overabundance contributes to neurodegeneration by promoting hyperactivation of the mTOR pathway, endoplasmic reticulum (ER) stress, autophagy dysfunction, RNA-protein condensates, amyloidogenesis, tau phosphorylation and neuronal apoptosis in cultured neurons and animal models of ALS, ALS/FTD and SCA2 (*C9orf72*, *Thy1*-TDP-43, and *ATXN2*^*Q127*^) [17–19, 23–25]. In the SCA2 mouse, decreasing *Stau1* by genetic interaction protects Purkinje cells, normalizes autophagy through mTOR and improves motor function [19, 20, 23]. Similarly, in the transgenic TDP-43 mouse spinal cord, reducing STAU1 normalizes autophagy by mitigating mTOR hyperactivity, increasing CHAT levels, and preventing glial activation and apoptosis [19, 23]. These findings demonstrate that STAU1 is a potential therapeutic target that could simultaneously improve multiple pathological features.

Because reducing pathological elevations in the RBP STAU1 is protective in multiple models of neurodegeneration, triggered by various mutations that affect different neuronal populations, we hypothesized that STAU1 could modulate the p53 pathway to prevent neuronal apoptosis. We examined the global transcriptomic landscape after *STAU1* knockdown (KD) and found the cellular immunity and apoptotic programs were inhibited, including the p53 pathway. In agreement, functional experiments in cultured human and mouse neurons, fibroblasts and SH-SY5Y cells demonstrated that *STAU1* KD prevented DNA damage and p53-mediated apoptosis induced by etoposide or Nutlin-3. In addition, in *C9orf72*-patient-derived fibroblasts and in vivo in a *C9orf72* expansion (BAC-C9-500) mouse, models of ALS/FTD, decreasing STAU1 was sufficient to prevent p53-mediated apoptotic signaling. Our findings reveal a novel role for STAU1 as a modulator of DNA damage, the p53 pathway, and apoptosis that could be harnessed to alter cell-fate decisions and prevent neurodegeneration.

## Results

### RNAseq highlights immunity and apoptosis pathways regulated by STAU1

Beginning with transcriptomic analysis in vitro, we examined the p53 pathway directly under conditions of STAU depletion in non-neuronal cells. This was followed by functional analysis in human neurons and finally in vivo using genetic interaction of ALS and STAU1-knockout (KO) mice.

**Fig. 1:**
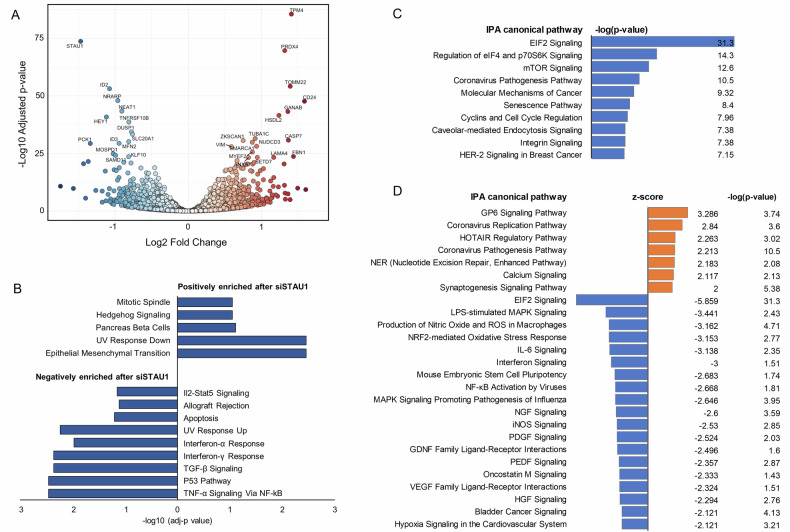
RNA-seq revealed a transcriptional signature of inhibition of apoptosis and immune responses by *STAU1* KD in HEK293 cells. **A** Volcano plot with highlighted DEGs. **B** Modified gene sets identified by GSEA-Hallmark pathway analysis. **C** Top 10 IPA canonical pathways ranked by *p*-value. **D** IPA canonical pathways with significant z-score and *p*-value, ranked by z-score.

We performed RNAseq in HEK293 cells after *STAU1* knock down with an siRNA (Fig. S1) and found a large number of differentially expressed genes (DEGs); 1644 transcripts were upregulated and 1466 transcripts were downregulated (adjP ≤ 0.1, log_2_ fold change (log_2_FC) cutoff of −0.1 and 0.1) (Fig. 1A). DEGs were within a log_2_FC range of −1.75 to 1.61. Hallmark pathway analysis [26] showed 5 pathways were positively enriched and 9 were negatively enriched after *STAU1* KD, composing a gene signature landscape of suppressed apoptotic and immune responses (Fig. 1B). These included multiple pathways that directly regulate immunity, cellular stress responses and death, including the epithelial-to-mesenchymal-transition, UV-response pathways, interferon-α and interferon-γ response, TGF-beta and TNF-alpha signaling via NF-kB, among others (Fig. 1B, Fig. S2A, B). In addition, both the p53 pathway and Apoptosis Hallmark gene sets were negatively enriched (Fig. 1B, Fig. S2A, B).

Interrogation of canonical pathways using the Ingenuity Pathway Analysis (IPA) software revealed 241 significant pathways (with *p* ≤ 0.05) (Fig. 1C for the top 10, Supplemental Table 1 for the complete list). In our previous studies we described how *STAU1* KD decreases the activity of eIF2 and mTOR, as well as their corresponding downstream targets [17–19]. In agreement with this, the top 3 IPA canonical pathways in the current analysis were *eIF2 signaling*, *regulation of eIF4 and p70S6K signaling* and *mTOR signaling* (Fig. 1C). IPA analysis of the DEGs in the context of their pathway assigns a positive z-score to the pathways that are predicted to be activated and a negative z-score to pathways predicted to be inhibited [27]. Out of 241 significant canonical pathways, IPA assigned significant z-scores (z-score ≥ 2 or ≤ -2) [27] to 26 pathways (Fig. 1D). The top 3 canonical pathways *eIF2 signaling*, *regulation of eIF4 and p70S6K signaling* and *mTOR signaling* had significant or strongly trending negative z-scores, mirroring our previous findings and indicating an accurate IPA prediction of their functional inhibition (−5.859, −1.886 and −1.732 correspondingly, Fig. 1D, Fig. S3, SI Table 1 and 2) [17–19].

**Fig. 2:**
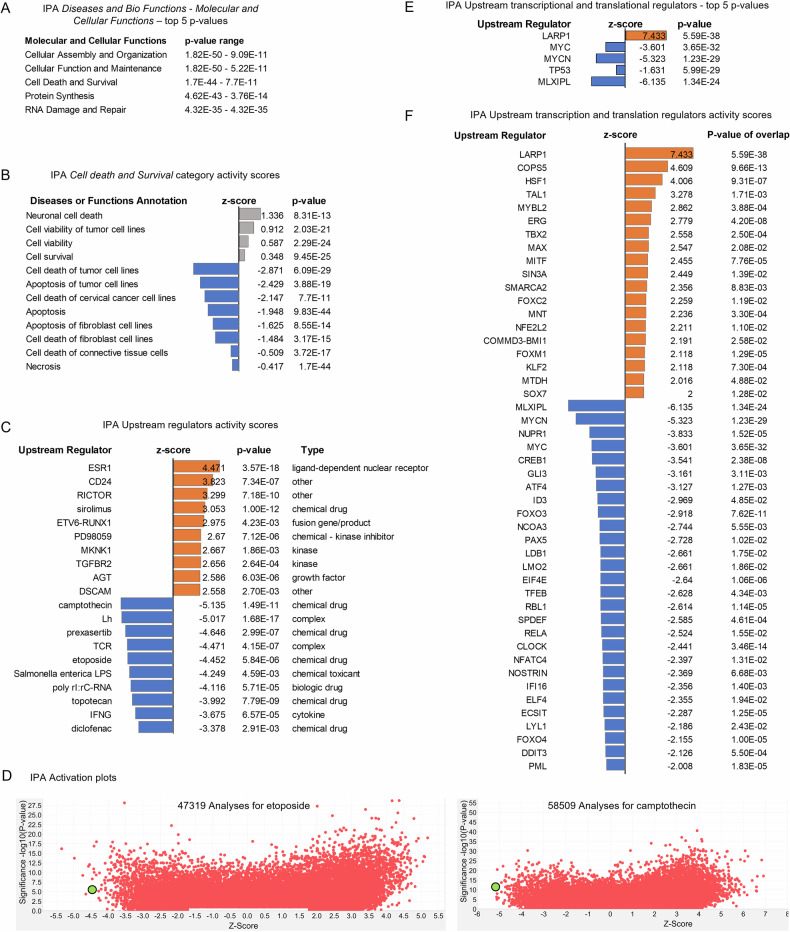
IPA functional and upstream analysis. **A** Top 5 IPA results for Diseases and Bio Functions - Molecular and Cellular Functions. **B** IPA results for “Cell death and Survival” category, ranked by *z*-score. **C** IPA Upstream analysis showing the top 10 significant positive and negative z-scores for upstream regulators, excluding transcriptional and translational regulators. **D** IPA Activation plots for etoposide and camptothecin. Red dots indicate a singular dataset from the IPA database and the green circle indicates our si*STAU1* dataset. **E** Top 5 IPA transcriptional and translational upstream regulators, ranked by *p*-value. **F** IPA upstream analysis showing all transcription and translation regulators with significant z-score.

Further analysis with IPA Diseases and Bio Functions highlighted that multiple Molecular and Cellular Functions were affected by *STAU1* KD (Fig. 2A). Of these, we studied the Cell Death and Survival category and found 3 annotations linked to cell death in different contexts that had significant negative z-scores, predicting their inhibited status (Fig. 2B). In addition, the Apoptosis annotation showed a trend towards inhibition, with a z-score of −1.948, just under the cutoff of −2. The canonical Apoptosis Signaling pathway contains 104 annotated molecules, out of which 45 were downregulated (43.3%) and 43 were upregulated (41.3%) in our dataset, and 16 (15.4%) not present.

IPA Upstream Regulator analysis identifies and predicts a positive or negative activation state for upstream transcription factors, microRNAs, kinases, compounds and drugs based on prior knowledge of their effects on the target genes in our dataset. Upstream analysis predicted an inhibition of the effects of multiple cytotoxic drugs with antineoplastic activity (camptothecin, prexasertib, etoposide and topotecan), along with a decrease in immune responses (TCR, Salmonella enterica LPS, poly rl:rc-rna, IFN-γ, diclofenac) (Fig. 2C). Several activated upstream regulators further support attenuation of cell death and immune responses by *STAU1* KD (ESR1, Rictor, sirolimus), however some can promote cell death and positively modulate immune responses, depending on the context (CD24, PD98059, Mknk1, Tgfbr2, DSCAM) (Fig. 2C). Activity plots for predicted responses to etoposide and camptothecin show the z-scores for these compounds in the context of >47,000 datasets from the IPA database, indicating *STAU1* KD has a profound effect (Fig. 2D).

The Upstream analysis of transcription and translation factors shows the top five factors with most significant *p*-values are MYC, MYCN and MLXIPL (belonging to the Myc/Max/Mad superfamily) and *TP53* and *LARP1*(Fig. 2E, F, Fig. S4). All these factors can be regulated by mTORC1, and STAU1 binds to the *mTOR* 5’-UTR and controls its abundance and activity [19]. In addition, multiple other factors with significant *p*-values and z-scores were identified as upstream regulators (Fig. 2F, SI Table 2).

Altogether, *STAU1* KD cells demonstrated extensive transcriptomic changes that suggest a cellular state of suppressed immune and apoptotic responses.

### Decreased STAU1 attenuates apoptosis

Because the transcriptomic analysis indicated *STAU1* KD could result in apoptotic resistance, we tested this hypothesis by treating cells with stressors that affect different cellular processes but converge at the activation of the intrinsic apoptosis pathway. We quantified cleaved Caspase-3 (cCaspase 3) and cleaved PARP (cPARP), as molecular effectors and markers of apoptosis [28, 29]. We utilized siRNA to KD *STAU1* in HEK293 cells and an AAV coding for a miRNA targeting *STAU1* in SH-SY5Y cells (AAV-PhP.eB-miSTAU1-45, due to low transfection efficiency), and additionally tested primary fibroblasts derived from *Stau1*-KO mice.

**Fig. 3:**
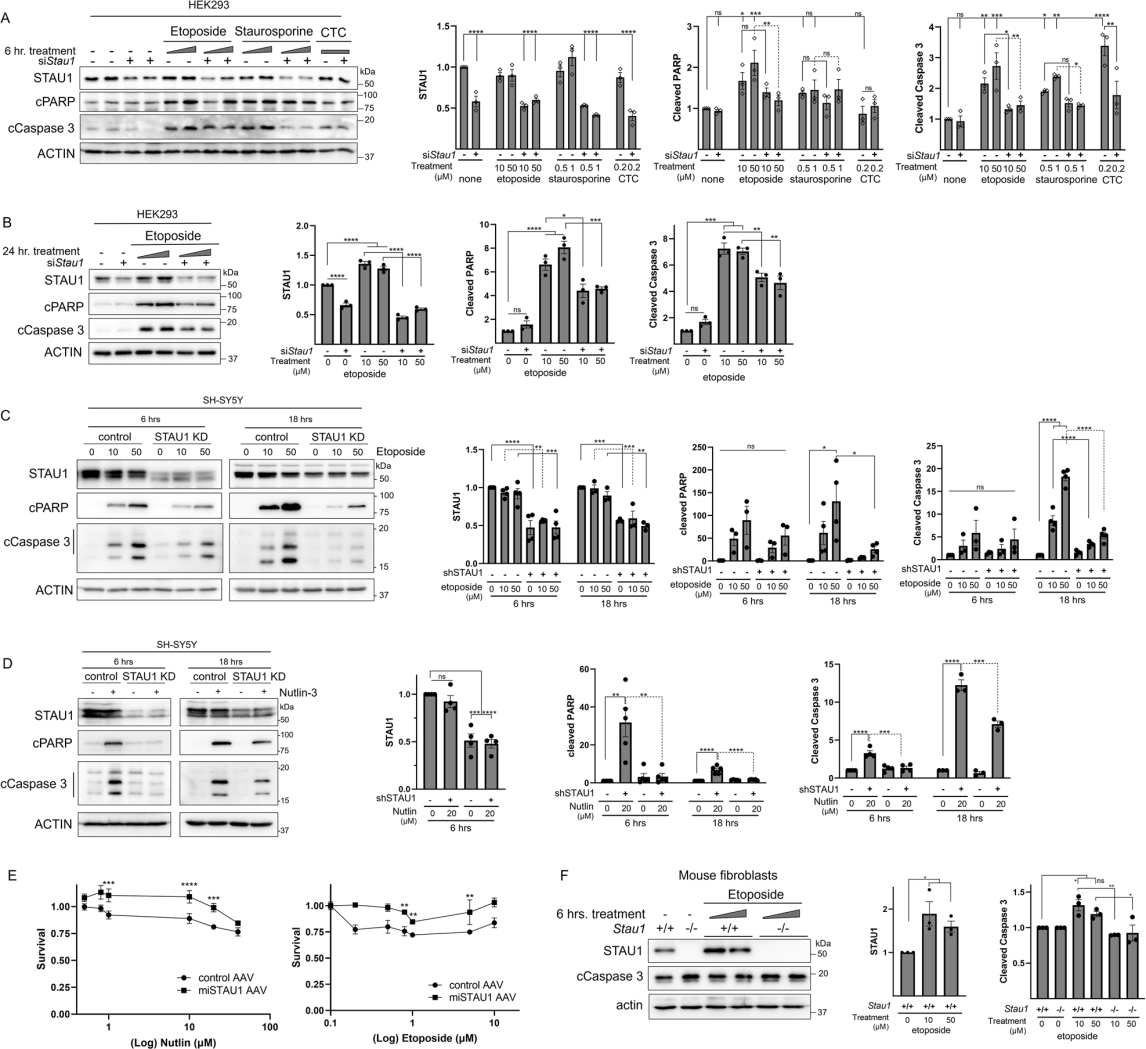
*STAU1* KD prevents apoptosis triggered by multiple stressors. **A**, **B** HEK293 cells were transfected with *STAU1* siRNA and after 48 h. treated with the indicated compounds. Apoptosis was evaluated by quantifying the increase in cleaved Caspase-3 (cCaspase 3) and cleaved PARP (cPARP) by western blot. **C**, **D** SH-SY5Y cells were transduced with an AAV expressing a miRNA against *STAU1* and treated with the indicated compounds after 36 h. **E** Cell viability of SH-SY5Y cells quantified with a fluorogenic Gly-Phe-AFC assay. **F** Western blots of primary fibroblasts from *Stau1*^+/+^ and *Stau1*^−/−^ mice treated with etoposide. Graphs in **A**–**E** correspond to western blot quantifications after 3 independent experiments and data are presented as mean ± SEM. Statistical analysis was performed by one-way ANOVA followed by Sidak’s multiple comparison test. *P ≤ 0.05, **P ≤ 0.01, ***P ≤ 0.001, ****P ≤ 0.0001.

After *STAU1* KD, HEK293 and SH-SY5Y cells were resistant to apoptosis triggered by Nutlin-3 (nut), staurosporine, etoposide (eto) and camptothecin (CTC), with levels of cCaspase 3 and cPARP comparable to untreated controls or highly attenuated (Fig. 3A–E). This protection was still present after 24 h of continuous exposure to etoposide (Fig. 3B) and cell viability was also preserved (Fig. 3E). In addition, in *Stau1* KO fibroblasts, etoposide did not elicit an apoptotic response (Fig. 3F). These results indicate that in the context of low STAU1, cells can evade apoptosis triggered by multiple stressors.

### Decreased STAU1 prevents DNA damage and pro-apoptotic p53 signaling

**Fig. 4:**
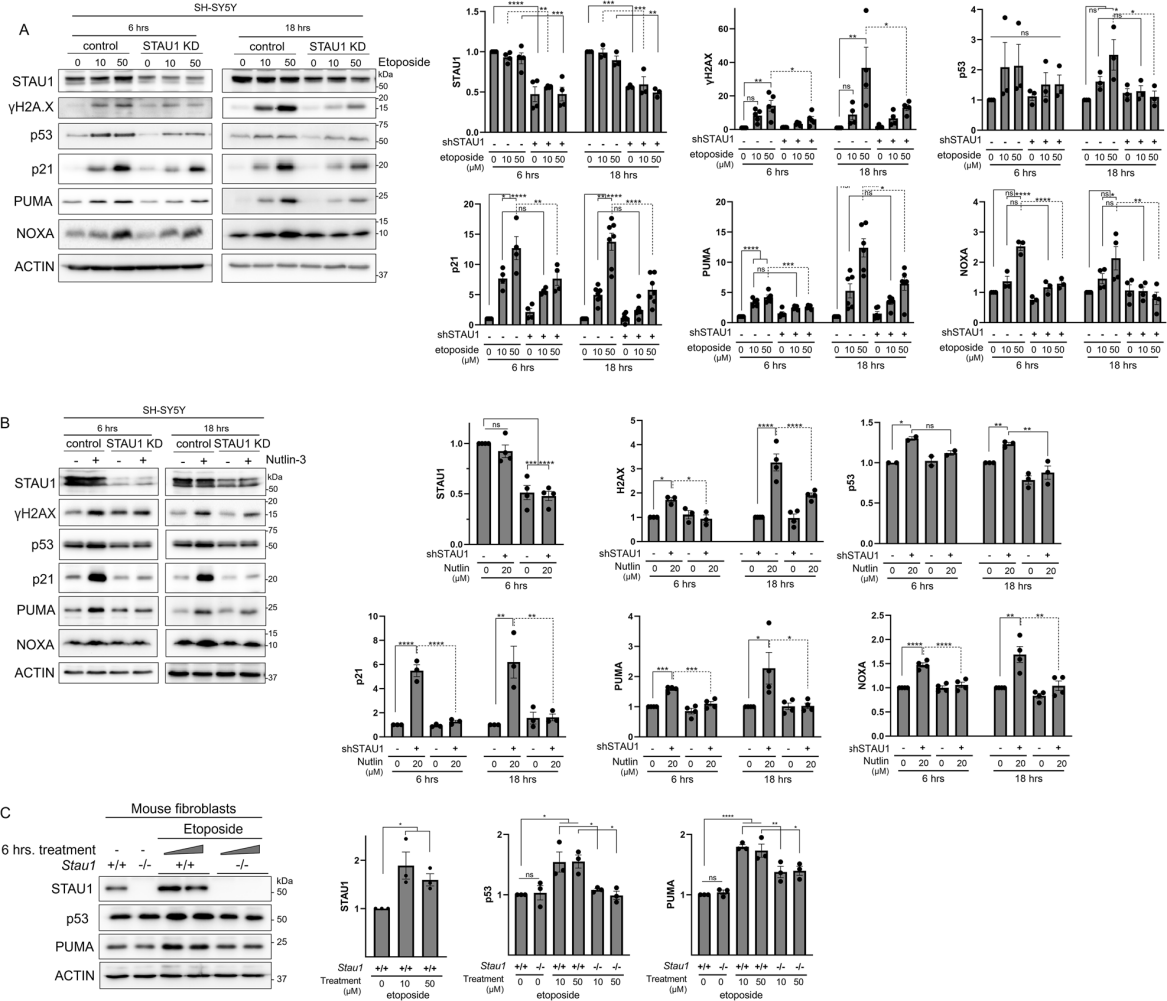
*STAU1* KD decreased p53 pro-apoptotic signaling. Western blots of SH-SY5Y cells treated with (**A**) etoposide (eto) or (**B**) Nutlin-3 (nut, 20 µM) for 6 or 18 h. **C** Western blots of primary fibroblasts from *Stau1*^+/+^ and *Stau1*^−/−^ mice (WT and *Stau1* KO) treated with etoposide 40 µM for 6 h. Graphs correspond to western blot quantifications after 3 independent experiments and data are presented as mean ± SEM. Statistical analysis was performed by one-way ANOVA followed by Sidak’s multiple comparison test. *P ≤ 0.05, **P ≤ 0.01, ***P ≤ 0.001, ****P ≤ 0.0001.

To study whether apoptotic resistance induced by *STAU1* KD was rooted in its modulation of the p53 pathway, we investigated p53 and apoptotic signaling triggered by etoposide and Nutlin-3. Etoposide induces DNA damage by inhibiting topoisomerase II [30], and Nutlin-3 is an MDM2 inhibitor that stabilizes and activates p53, and also causes DNA damage [31, 32]. Upon DNA damage, the histone H2AX is rapidly phosphorylated to γH2AX, triggering the DNA damage response and recruiting repair machinery to the sites of damaged chromatin [30, 31]. Both etoposide and Nutlin-3 caused DNA damage, evidenced by an increase in γH2AX, and *STAU1* KD completely prevented or decreased its induction (Fig. 4A, B). *STAU1* KD also lowered p53, its target gene p21 and PUMA and NOXA, mediators of apoptosis downstream of p53 activation. To test whether the activation of the DDR contributes to apoptotic resistance in iNeurons, we evaluated the abundance of phospho-ATM and phospho-DNA-PKcs by western blot (Fig. S5). After STAU1 knockdown, these mediators decreased following etoposide and increased after Nutlin-3, suggesting a functional DNA damage response that may support neuronal survival in a context-dependent manner (Fig. S5).

We also carried out a Comet assay, which detects DNA damage at the single cell level. Due to the assay’s high sensitivity, our cells’ baseline measurements after transfection left us with a narrow dynamic range and we were not able to meaningfully quantify DNA damage changes with this technique (Fig. S6).

To further support our findings, we studied primary skin fibroblast cultures from WT and *Stau1* KO mice [33]. In WT fibroblasts, etoposide caused p53 stabilization and increased PUMA, associated with increased cCaspase 3, whereas in *Stau1*^−/−^ fibroblasts their levels were indistinguishable from untreated cells (Fig. 4C).

### STAU1 reduction blocks DNA damage and p53-driven apoptosis in neurons

**Fig. 5:**
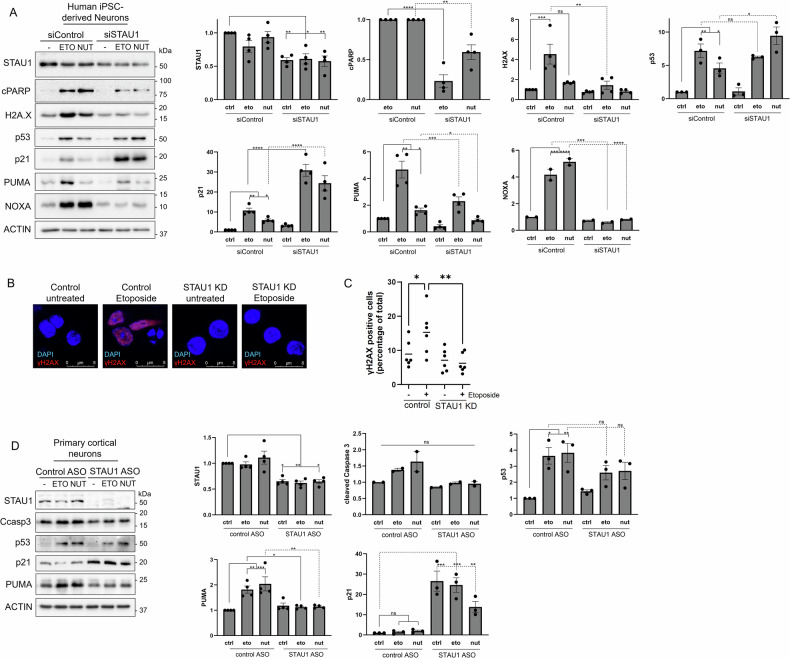
*STAU1* KD prevents neuronal apoptosis mediated by p53. **A** Western blots of human induced neurons (iNeurons) transfected with a control or *STAU1* siRNA twice (siControl, siSTAU1) and 48 h later treated with etoposide (eto, 40 µM) or Nutlin-3 (nut, 20 µM) for 6 h. cPARP was quantified normalized to a treated control because the levels in untreated cells were undetectable. **B** Ilustrative photographs of iNeuron nuclei immunostained for γH2AX. **C** Quantification of nuclei immunopositive for γH2AX in each condition. >300 nuclei per condition were counted, in 6 different fields from 3 independent cultures **D** Western blots of primary mouse cortical neurons in culture with a control ASO or an ASO targeting *STAU1*. Graphs correspond to western blot quantifications after 3 independent experiments and data are presented as mean ± SEM. Statistical analysis was performed by one-way ANOVA followed by Sidak’s multiple comparison test. *P ≤ 0.05, **P ≤ 0.01, ***P ≤ 0.001, ****P ≤ 0.0001.

Because STAU1 is increased in neurodegeneration associated with mutations in *TDP-43*, *MAPT*, *PSEN1*, *C9orf72* and *HTT* [14, 16–23], we investigated whether targeting *STAU1* could prevent neuronal apoptosis. We studied neurons differentiated by inducible overexpression of NGN-2 (induced Neurons – iNeurons) (Fig. S7) [34–36] and found that *STAU1* KD reduced DNA damage and p53-dependent apoptosis (cPARP, γH2AX, PUMA and NOXA) after exposure to etoposide and Nutlin-3 (Fig. 5A). Immunostaining of iNeurons for γH2AX and quantification of positive nuclei also evidenced prevention of DNA damage by *STAU1* KD (Fig. 5B, C).

In contrast with SH-SY5Y cells and fibroblasts, siSTAU1 did not reduce p53 activation in neurons; etoposide and Nutlin-3 induced p53 to the same level or higher (correspondingly) in control and *STAU1* KD neurons. At the same time, induction of p21 in response to etoposide and Nutlin-3 was higher in the *STAU1* KD neurons. The protein p21 is a key target of p53, and its activation promotes cell cycle arrest, DNA repair and inhibition of apoptosis [37–39]. In addition to iNeurons, we studied primary mouse cortical neurons and found they mirrored the iNeuron results, with an increase in p21 after STAU1 KD (Fig. 5B). These results demonstrate that STAU1 KD can prevent DNA damage and p53-dependent apoptosis by harnessing the signaling pathway in a cell type and context-dependent manner.

### Decreased *STAU1* prevents p53 pro-apoptotic signaling triggered by *C9orf72* mutation

**Fig. 6:**
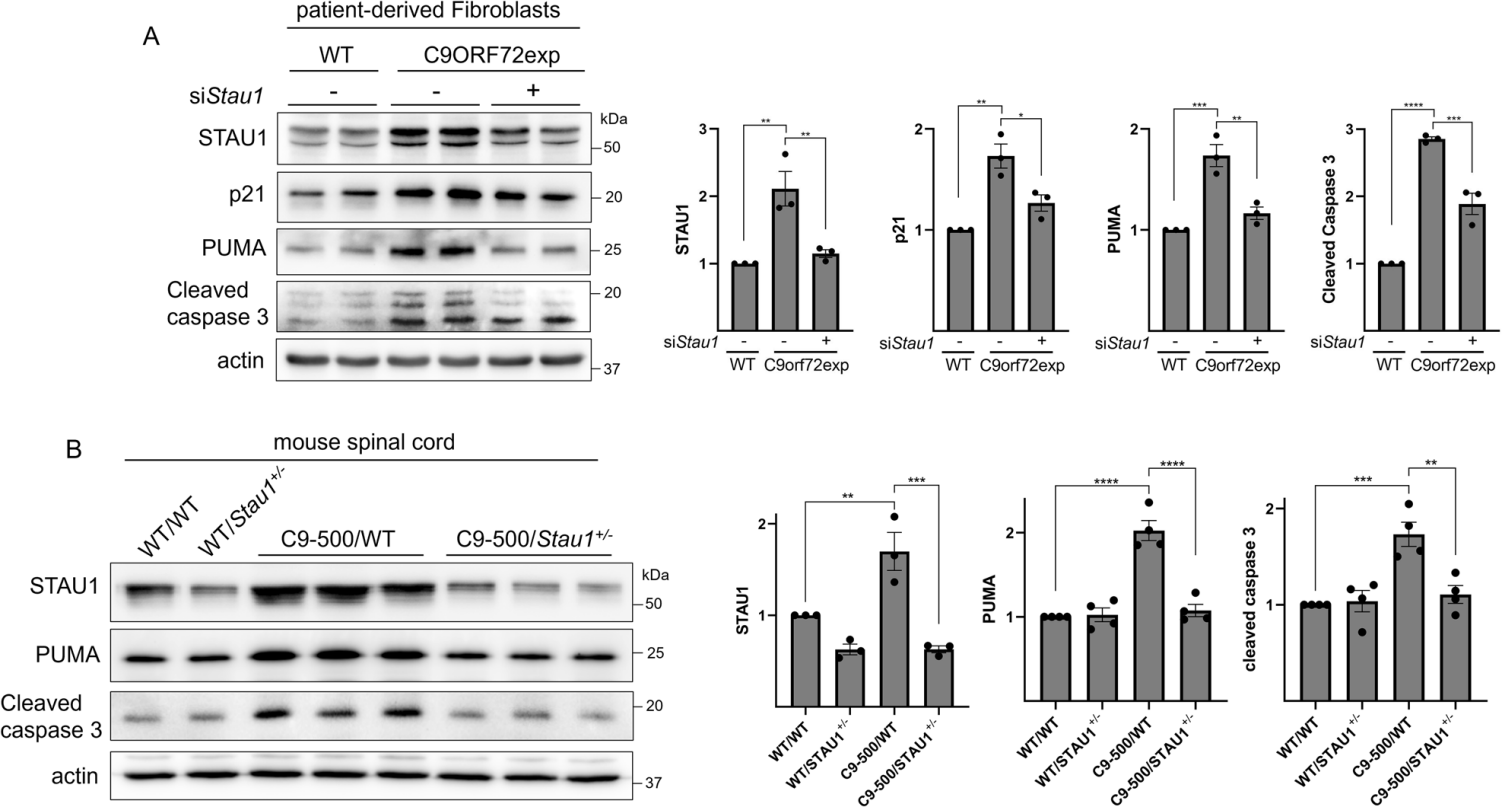
Reducing STAU1 levels prevents pro-apoptotic p53 signaling and attenuates apoptosis triggered by *C9orf72* expansion. **A** Western blots of patient-derived fibroblasts expressing a *C9orf72* expansion and corresponding quantification graphs. **B** Western blots of spinal cord lysates from *C9orf72*-500 mice crossed with *Stau1*^-/−^ mice, and corresponding quantification graphs. Graphs correspond to western blot quantifications after 3 independent experiments and data are presented as mean ± SEM. Statistical analysis was performed by one-way ANOVA followed by Sidak’s multiple comparison test. *P ≤ 0.05, **P ≤ 0.01, ***P ≤ 0.001, ****P ≤ 0.0001.

*C9orf72*-exp triggers DNA damage and p53-PUMA dependent neuronal apoptosis in cellular, fly and mouse models [40–42]. In addition, STAU1 is highly overabundant in fibroblasts, neurons, mice, and human post-mortem spinal cord tissues with ALS/FTD-linked *C9orf72* pathological expansions (*C9orf72*-exp) [17–19]. We therefore hypothesized that *STAU1* KD could prevent p53-mediated apoptosis in models of *C9orf72*-exp toxicity [40]. We utilized patient-derived fibroblasts expressing *C9orf72*-exp and found they expressed high levels of p21, PUMA and cCaspase-3 at baseline (Fig. 6A). p53 was below our level of detection in these cells, which is often the case in unstimulated, non-transformed cells, although the increase in p21 and PUMA provided evidence for activation of the p53 pathway [37, 38]. In these cells, lowering STAU1 decreased p21, PUMA and cCaspase-3 caused by *C9orf72-*exp (Fig. 6A).

To investigate whether STAU1 can modulate the p53 pathway to prevent apoptosis in vivo we utilized the *C9orf72*-500 mouse model of ALS/FTD [43]. We found that in spinal cords, STAU1, PUMA and cCaspase-3 were elevated (Fig. 6B). When crossed with *Stau1*^*−/−*^ mice to produce *C9orf72*-500 mice haploinsufficient for *Stau1*, both PUMA and cCaspase-3 were reduced to levels comparable to WT mice (Fig. 6B). Thus, genetic reduction of *Stau1* was sufficient to prevent p53-mediated apoptotic signaling in a mouse model of neurodegeneration caused by *C9orf72-*exp. Altogether, our results indicate reducing STAU1 abundance can effectively prevent p53 apoptotic signaling to protect from apoptosis in human and mouse cellular models and in vivo.

## Discussion

The p53 pathway is one of the major apoptotic signaling pathways and mounting evidence of its involvement in neurodegeneration positions it as a target for maintaining neuronal function and viability in multiple diseases. In this study, we identified a novel role for the RBP STAU1 in preventing DNA damage and p53-mediated apoptosis. With transcriptomic and functional analyses, we determined that in conditions of low STAU1 abundance, cells are resistant to apoptosis triggered by activation of p53, either by DNA damage or p53 stabilization (etoposide and Nutlin-3). We evidenced this in cell lines, human and mouse neurons in culture and in patient-derived fibroblasts and mice with *C9orf72* expansions, models of ALS/FTD. These findings add the prevention of p53-mediated apoptosis to the multiple mechanisms of neuroprotection mediated by STAU1 depletion already described [17–20].

The transcriptomic landscape of *STAU1* KD cells indicated the acquisition of an anti-inflammatory and apoptotic resistant state, and identified multiple pathways and upstream regulators by which STAU1 could modulate p53-mediated apoptosis. These included the mTOR and PERK/p-eIF2α/CHOP pathways, which are harnessed by STAU1 in a bidirectional manner to either promote or prevent apoptosis [17–20]. IPA and hallmark pathway analysis also predicted extrinsic apoptosis and immunity pathways to be functionally inhibited in response to *STAU1* KD, mainly TGF-β, TNF-α, Nf-kB and Interferon response pathways. This could determine that *STAU1* KD would not only prevent apoptosis triggered by intrinsic stress, but also in response to environmental factors and extracellular signaling. In neurological conditions, the inhibition of these pathways is predicted to limit the spread of neuroinflammation and non-cell autonomous neuronal death, whereas in cancer, evasion of immunosurveillance and apoptotic resistance would constitute a risk. Despite this, no propensity to tumorigenesis has been described in *STAU1* KO mice [33, 44].

Transcriptional analysis placed the transcription factors MYC, MYCN, and MLXIPL (all belonging to the MYC/MAX/MAD superfamily) within the top 4 most likely functionally inhibited transcription factors. Canonically, c-MYC is required for efficient induction of p53-dependent apoptosis after DNA damage [45]. Previous studies have shown that STAU1 can interact with the 5’UTR of c-MYC to enhance its translation, increasing its mRNA and protein abundance, along with STAU1 KD decreasing c-MYC levels [46, 47]. Low c-MYC abundance promotes apoptotic resistance, while high levels increase apoptosis by repressing p21. This scenario mirrors *STAU1* KD in neurons, with prevention of p53-mediated apoptosis associated with a p21 increase. It is therefore plausible that *STAU1* KD could induce apoptotic resistance by decreasing c-MYC through its functions as an RBP.

While it is not a direct measurement of DNA damage, phosphorylation of Histone H2AX (γH2AX) at sites of DNA damage is one of the earliest detectable events, and it rapidly activates the DNA damage response (DDR) and checkpoint-mediated cell cycle arrest, serving as a widely accepted surrogate marker [48, 49]. After *STAU1* KD, etoposide induced little to no γH2AX increase, suggesting *STAU1* KD conferred protection against genotoxic stress. In agreement with this, transcriptome pathway analysis identified the *nucleotide excision repair (NER)* as functionally activated and *UV response* genes were enriched, indicating an enhancement in DNA repair pathways. In addition, IPA upstream-regulators analysis predicted an inhibited response to the genotoxic drugs etoposide and camptothecin. These results indicate *STAU1* KD leads to transcriptomic changes that determine resistance to DNA damage, however, whether this mechanism is directly involved in preventing apoptosis remains to be studied.

STAU1 binds to different types of STAU1-binding sites (SBS) in double stranded RNA. Binding to the 5’UTR SBS or specific coding regions can enhance the translation of the mRNA while binding to the 3’UTR recruits UPF-1 to trigger Staufen-mediated decay (SMD) degradation of the mRNA or prevent its translation [44, 50–55]. Through these functions STAU1 can post-transcriptionally regulate a multitude of target mRNAs, affecting numerous cellular networks.

After DNA damage or Nutlin-3 exposure, p53 protein abundance rapidly increases and activates downstream transcriptional targets that will determine cell fate. We found that in dividing cells, *STAU1* KD prevented the increase of p53 and downstream apoptotic effectors. In contrast, in human and mouse neurons, *STAU1* KD did not decrease p53 levels, but its signaling, however, shifted from apoptotic to pro-survival, inducing p21. The protein p21 is a major target of p53, preventing neuronal death by blocking cell cycle re-entry signaling, promoting DNA repair, reducing oxidative stress and inhibiting caspase-3, among other homeostatic functions [38–40]. Therefore, it is likely that the neuroprotective effect of *STAU1* KD is mediated by fine-tuning p53 signaling and its downstream effectors, shifting the therapeutic paradigm of achieving neuroprotection by merely reducing p53 levels. However, whether *STAU1* KD causes sustained p21 activation and subsequent neuronal senescence remains to be investigated. [37–39, 56]. Despite the mechanistic differences, *STAU1* KD promoted survival in all cell types studied. These results indicate that blocking the activation of the p53 pathway is not required for *STAU1* KD to prevent p53-mediated apoptosis; and that STAU1 is likely to act upstream of the p53 pathway, leading to cell- and context-dependent responses that determine apoptotic resistance.

The accumulation of DNA damage and activation of its associated signaling pathways are now recognized as major drivers of neurodegeneration and brain aging. Dividing glial cells are highly susceptible to DNA damage during replication, whereas post-mitotic neurons must maintain their genomic integrity throughout their lifespan. In dividing cells, *STAU1* KD could potentially protect against this damage by triggering cell cycle arrest. However, the effect of reducing STAU1 levels varies by cell type—it impairs proliferation in non-transformed cells but does not affect transformed cells [57, 58]. In our study, we examined HEK293 cells, SH-SY5Y cells, mouse primary cortical neurons, iPSC-derived neurons, and patient-derived fibroblasts, encompassing transformed, non-transformed, and post-mitotic cells. Regardless of cell type and mitotic status, lowering STAU1 consistently reduced γH2AX levels and apoptosis. These findings suggest that the protective mechanisms that we observed are independent of the cell cycle.

Pathological increases in STAU1 are observed across the nervous system in multiple models of neurodegeneration, triggering ER stress, autophagy defects and neurodegeneration [17–20, 23]. In post-mortem spinal cords of sporadic and *C9orf72-*ALS patients and muscle of DM1 patients, STAU1 is also highly overabundant [17, 59]. A survey of patient-derived fibroblasts with mutations in *TDP-43*, *MAPT*, *PSEN1*, *C9orf72* and *HTT* found increased STAU1 in all [18–20]. Likewise, in cellular and animal models of ALS/FTD (TDP-43 and *C9orf72*) and SCA2 (*ATXN2-*Q127), decreasing *STAU1* by RNAi or genetic interaction reverses molecular markers of ER stress, defective autophagy, protein aggregates, neuronal death and glial activation, and improves the behavioral motor phenotype of SCA2 mice [17–19]. In addition, we now show decreasing STAU1 can prevent DNA damage and neurodegeneration mediated by the p53 pathway.

A growing body of evidence implicates DNA damage and p53 in the pathogenesis of ALS/FTD [60, 61]. P53 is strongly activated in *C9orf72*, *TARDBP* and sporadic ALS/FTD post-mortem tissue, iPSC-motor neurons and animal models [4–6, 8, 40, 62–66]. Nuclear TDP-43 depletion, knockdown or overexpression causes DNA damage and robust p53 upregulation [4, 40, 64, 67–69]. DNA damage, somatic mutations, gene fusions, the DNA damage response and p53 activation are established hallmarks of neurodegeneration caused by *C9orf72* repeat expansions in ALS/FTD [4–9]. *C9orf72*-toxicity triggers p53-PUMA dependent neurodegeneration, and in a *C9orf72* mouse model (expressing the dipeptide repeat poly(PR)) ablating p53 completely prevents neuronal death and extends their lifespan [40]. In agreement with this, we found patient-derived fibroblasts with *C9orf72*-exp had increased levels of PUMA, p21 and cleaved caspase 3, which *STAU1* KD normalized. In the *C9orf72-*500 mouse spinal cord, PUMA and cleaved caspase 3 were also increased, and heterozygosity for *Stau1* was sufficient to normalize them, demonstrating that STAU1 can prevent p53 apoptotic signaling in ALS/FTD in vivo.

Our findings show that STAU1 reduction results in protection against DNA damage and resistance to apoptosis induced by p53 in neurodegeneration. Because STAU1 is ubiquitously expressed and is increased in numerous neurodegenerative diseases and by acute stress, our findings constitute a novel mechanism to modulate p53-controlled cell fates that could be harnessed to prevent neuronal stress and apoptosis.

## Materials and methods

A detailed list of all materials, experimental models and software can be found in SI Table 3.

### Cell culture, transfection, transduction and treatment

Cell culture media and reagents were purchased from Thermo Fisher Scientific unless otherwise specified. HEK293 cells, mouse skin fibroblasts and human skin fibroblasts were grown in DMEM supplemented with 10% fetal bovine serum and 1X penicillin and streptomycin.

Mouse primary fibroblasts were obtained by culturing ~3 mm skin explants attached to a cell culture dish for 2–3 weeks. Explants were then removed and fibroblasts were passaged 2–3 times before experimentation.

Human fibroblasts ND38530 (normal) and ND42506 (GGGGCC repeat expansion>24; in 1st intron – individual at risk for FTD) were obtained from the Coriell Cell Repositories (Camden, NJ, USA).

Identity authentication of HEK293, SH-SY5Y cells and human fibroblasts was carried out by short tandem repeat (STR) analysis with the GenePrint 24 System (Promega, USA) and PCR mycoplasma testing was carried out regularly.

Lipofectamine 2000 was used for all siRNA and plasmid transfections, according to the manufacturer’s instructions. *STAU1* siRNA was used at 100 μM, and subsequent pharmacological treatments were added 48 h after transfection in fresh media.

### Primary culture of cortical neurons

Mice were bred and handled according to the protocol for ethical use of animals for research approved by the University of Utah IACUC (protocol number 00002040). Primary cortical neuron cultures were prepared from neonatal mice [70, 71]. Brain cortices from 6–7 animals were isolated and incubated with 50 units of papain (Worthington) in Earle’s balanced salt solution (EBSS) with 1mM L-cysteine and 0.5 mM EDTA for 15 min at 37 °C, followed by washing in EBSS and mechanical trituration. The remaining tissue was removed by filtration through a 50 µm strainer (Falcon). Neurons were seeded at 50,000 per cm^2^ on plates coated with poly-L-ornithine and laminin in Neurobasal Plus medium containing 2% B27 Plus supplement (Life technologies) and glutamax 0.5 mM. On day 2 10 µM cytosine arabinoside was added for 24 h to prevent proliferation of glial cells and 75% of culture medium volume was replenished every 2–3 days from there on. After 2 weeks in culture, we added ASO against *STAU1* or a non-targeting control ASO for overnight gymnotic delivery. Neurons were treated 48 h later.

### Culture and differentiation of iPSCs

The iPSC line KOLF2.1 J AAVS-TO-NGN2 was a gift from Michael Ward (NIH, Maryland) and Bill Skarnes (Jackson laboratories, Connecticut) as part of the iPSC Neurodegenerative Disease Initiative (iNDI) and has been previously validated [72]. iPSCs were cultured on Geltrex substrate with StemFlex cell culture media. For differentiation, iPSCs were passaged using Accutase (Sigma) and plated at high density on Geltrex substate. For the first 3 days cells were grown in Induction Media composed of DMEM/F12 supplemented with 1% N2, 1% NEAA and 1% GlutaMax, with the addition of 2 μg/mL doxycycline (Sigma), with daily media changes. On day 3 cells were dissociated with Accutase and plated on Geltrex substrate in 12 well plates at a density of 5 × 10^4^ cells per well. The next day, media was changed to Maturation Media, composed of Neurobasal Plus supplemented with 1% B27 Plus, 1% N2, 10 ng/mL BDNF (StemCell) and 2 μg/mL doxycycline. Doxycycline was discontinued on day 8. 50% of the media was replaced with fresh media 2-3 times per week. iN from at least two different batches of differentiation were used for experiments. iN were transfected between days 15 and 20 after initial induction with Lipofectamine RNAiMAX and 400 nM siSTAU1 or siControl, overnight, for 2 consecutive days, and experiments were carried out 48 h after the last transfection.

### Immunofluorescence

For immunofluorescence analysis iNeurons were plated on coverslips coated with geltrex on day 3 of differentiation, transfected on days 4 and 5 and on day 7 treated with etoposide for 2 h. iNeurons were pre-fixed by adding 4% paraformaldehyde into the cell culture media and incubating for 15 min, then fixed for further 15 minutes by replacing the entire volume of media with paraformaldehyde 4%. iNeurons were permeabilized and blocked for 1 h with 5% goat serum and 0.1 Triton X-100 in PBS, then incubated with γH2AX antibody at a 1:500 dilution overnight. The secondary antibody was an Alexa Fluor Plus 594 used at a 1:1000 dilution for 1 h. Images were acquired with a Nikon widefield microscope or a Leica SP8 at the Cell imaging core of the University of Utah.

### Construction of AAV vector and production of viral particles

The plasmids used to generate adeno associate virus (AAV) include the single-strand AAV expression plasmid, pAAV-U6-sgRNA-CMV-GFP, (gift from Hetian Lei; Addgene plasmid # 85451), and pUCmini-iCAP-PHP.eB, (gift from Viviana Gradinaru; Addgene, plasmid # 103005), and pHelper (Stratagene, La Jolla, CA, USA). The STAU1 miRNA DNA insert “CGAGTGAGCGCTAACTGCCATGATAGCCCGAGCTGTAAAGCCACAGATGGGCTCGGGCTATCATGGCAGTTACCGCCTACTATTTTTTA” was cloned downstream of the U6 promoter of sgRNA pre-depleted AAV-U6-CMV-GFP plasmid using Sac I and Spe I restriction sites and designated as pAAV-U6-iSTAU1-45F(h + m)-CMV-GFP. The plasmid constructs were verified by sequencing. Recombinant AAV particles were generated as previously described [73] with minor modifications. HEK293T cells were co-transfected with three plasmids: either pAAV-U6-CMV-GFP (control) or pAAV-U6-iSTAU1-45F(h + m)-CMV-GFP along with pUCmini-iCAP-PHP.eB and pHelper, at a ratio of 1:4:2 (based on micrograms of DNA; ~ 30 μg of total DNA per 10 cm dish) using lipofectamine 2000 transfection reagent (ThermoFisher Scientific) according to the manufacturer’s protocol. After 12–18 h media was changed and incubated for further 72 h. Cells were then harvested and lysed in 25 mM Tris, pH 8.5, 150 mM NaCl with repeated freezing and thawing cycles using liquid nitrogen and a 37 °C water bath, followed by centrifugation at 4 °C for 30 min at 14,000 rpm. The resultant supernatants containing AAV-PhP.eB-Control or AAV-PhP.eB-miSTAU1-45 were filtered and stored at −80 C. SH-SY5Y cells were incubated overnight with these AAVs in a 1:4 dilution with fresh media, and experiments were conducted 48 h later.

### Viability assay

For quantification of cell viability, we used the Cell-Titer Fluor Viability Assay (Promega), a glycyl-phenylalanyl-aminofluorocoumarin based-assay. SH-SY5Y cells were plated in black 96 well plates with optical bottoms, and the assay was carried out according to the manufacturer’s protocol.

### Mice

All mice were housed and bred in standard vivarium conditions and experimental procedures were approved by the Institutional Animal Care and Use Committee (IACUC) of the University of Utah. The Stau1^tm1Apa(*−/−*)^ (*Stau1*^*−/−*^*)* mouse was a generous gift from Prof. Michael A. Kiebler, Ludwig Maximilian University of Munich, Germany and genotyped according to published protocols [33]. *Stau1*^*−/−*^ mice were maintained in a C57BL/6 J background.

FVB/NJ-Tg(*C9orf72*)500Lpwr/J mice [43] were purchased from JAX Laboratories and backcrossed to C57BL/6 J. C9-500 mice were first genotyped with a multiplex PCR amplifying mouse actin with JAX primers 21238 F and 21239 R, and human *C9orf72* primers amplifying across exon 10 (C9Ex10F) and exon 11 (C9Ex11R) producing a 415 bp and 320 bp amplicon, respectively. PCR was performed with an initial denaturation at 95 °C for 5’, 30 cycles of 95 °C for 1’, 60 °C for 30” and 72 °C 1’ with a final extension of 72 °C for 5’. The amplicon was run on a 3% agarose gel and detected with ethidium bromide. The G4C2 repeat expansion was then verified utilizing previously published Repeat Prime PCR protocol [74]. Only animals verified to be non-mosaic and with a repeat expansion greater than 200 bp were utilized. C9-500 mice were crossed with *Stau1*^*−/−*^ mouse to generate *C9-500/Tg Stau1*^*(+/−)*^, *C9-500/Wt Stau1*^*(+/−)*^, *C9-500/Tg Stau1*^*(+/+)*^ and *C9-500/Wt Stau1*^*(+/+)*^ in a mixed background of FVB/NJ and C57BL/6 J.

### RNA sequencing and analysis

Total RNA was extracted from tissues using the RNeasy Mini-Kit (Qiagen) according to the manufacturer’s protocol. RNA quality was determined using the Agilent ScreenTape Assay. Library preparation was performed using the Illumina TruSeq Stranded Total RNA library prep Ribo-Zero gold. Paired-end 150 bp reads were generated on a Novaseq 6000 S2 cell sequencing instrument at the High-Throughput Genomics and Bioinformatic Analysis Shared Resource at Huntsman Cancer Institute (University of Utah). The human GRCh38 genome and gene annotation files were downloaded from Ensembl release 100 and a reference database was created using STAR version 2.7.3a with splice junctions optimized for 150 base pair reads [75]. Optical duplicates were removed from the paired end FASTQ files using clumpify v38.34 [76] and reads were trimmed of adapters using cutadapt 1.16 [77]. The trimmed reads were aligned to the reference database using STAR in two pass mode to output a BAM file sorted by coordinates. Mapped reads were assigned to annotated genes using featureCounts version 1.6.3 [78]. The output files from cutadapt, FastQC, FastQ Screen, Picard CollectRnaSeqMetrics, STAR and featureCounts were summarized using MultiQC to check for any sample outliers [79]. Differentially expressed genes were identified for siSTAU1 vs siControl using a 5% false discovery rate with DESeq2 version 1.26.0 [80]. Pathways were analyzed using the fast gene set enrichment package with a 10% false discovery rate [81]. For IPA, we used a 5% false discovery rate and a log_2_FC cutoff of -0.1 and 0.1.

### Western blot

Protein homogenates from cultured cells were prepared by scraping cells in phosphate buffered saline and lysing the pellets in Laemmli sample buffer (Bio-Rad), followed by boiling for 5 min [82]. HEK293 protein extracts for detection of p53 were diluted 1:10. Mouse spinal cords were hydraulically ejected and protein extracts were prepared by homogenization in extraction buffer (25 mM Tris-HCl pH 7.6, 300 mM NaCl, 0.5% Nonidet P-40, 2 mM EDTA, 2 mM MgCl2, 0.5 M urea and protease inhibitors) followed by centrifugation at 4 °C for 20 min at 14,000 RPM. Supernatants were then diluted in laemmli sample buffer and boiled.

Proteins were resolved by SDS-PAGE and transferred to Hybond P membrane (Amersham Bioscience), blocked in Tris-buffered saline 0.1% Tween-20 with 5% skim milk and primary antibody was incubated overnight in this same solution. After incubation with the corresponding secondary antibody signal was detected using Immobilon Western Chemiluminescent HRP Substrate (EMD Millipore) or SuperSignal™ West Pico PLUS Chemiluminescent Substrate (ThermoFisher Scientific) and photographed with a Bio-Rad ChemiDoc. Analysis and quantification were performed with Image Lab software (Bio-Rad). Relative protein abundance was first normalized against actin band intensity and then expressed as the ratio to the normalized control.

### Comet assay

We used the *CometAssay Single Cell Gel Electrophoresis Assay* kit from BioTechne, according to the manufacturer’s instructions. The percentage of DNA in tail was quantified in ImageJ with the OpenComet *p*lug-in [83].

### Statistical analysis

All results are presented as mean ± standard error of the mean (SEM) unless noted otherwise. For western blot quantifications 3 independent experiments were carried out and the means for each group were compared with an Ordinary one-way ANOVA followed by Sidak’s multiple comparison test in the GraphPad Prism software.

### Ethics approval and consent to participate

All mice were housed and bred in standard vivarium conditions and experimental procedures were approved by the Institutional Animal Care and Use Committee (IACUC) of the University of Utah under protocol number 16-09007.

The KOLF2.1 J AAVS-TO-NGN2 cell line was obtained from Dr. Michael Ward and Dr. William Skarnes. The parental KOLF2.1 J iPSC line was produced according to all regulations and informed consent of the NRES Committee Yorkshire & The Humber Leeds West, approval number 15/YH/0391.

## Supplementary information

Supplemental material

Table 3

Full length uncropped original Western blots

## Data Availability

All data generated or analyzed during this study are included in this published article and its supplementary information files.

## References

[1] Abate G, Frisoni GB, Bourdon JC, Piccirella S, Memo M, Uberti D. The pleiotropic role of p53 in functional/dysfunctional neurons: focus on pathogenesis and diagnosis of Alzheimer’s disease. Alzheimers Res Ther. 2020;12:160. 33272326 10.1186/s13195-020-00732-0 PMC7712978

[2] Hollville E, Romero SE, Deshmukh M. Apoptotic cell death regulation in neurons. FEBS J. 2019;286:3276–98. 31230407 10.1111/febs.14970 PMC6718311

[3] Ranganathan S, Bowser R. p53 and cell cycle proteins participate in spinal motor neuron cell death in ALS. Open Pathol J. 2010;4:11–22. 21572928 10.2174/1874375701004010011 PMC3092395

[4] Ziff OJ, Neeves J, Mitchell J, Tyzack G, Martinez-Ruiz C, Luisier R, et al. Integrated transcriptome landscape of ALS identifies genome instability linked to TDP-43 pathology. Nat Commun. 2023;14:2176. 37080969 10.1038/s41467-023-37630-6 PMC10119258

[5] Farg MA, Konopka A, Soo KY, Ito D, Atkin JD. The DNA damage response (DDR) is induced by the C9orf72 repeat expansion in amyotrophic lateral sclerosis. Hum Mol Genet. 2017;26:2882–96. 28481984 10.1093/hmg/ddx170

[6] Lopez-Gonzalez R, Lu Y, Gendron TF, Karydas A, Tran H, Yang D, et al. Poly(GR) in C9ORF72-related ALS/FTD compromises mitochondrial function and increases oxidative stress and DNA damage in iPSC-derived motor neurons. Neuron. 2016;92:383–91. 27720481 10.1016/j.neuron.2016.09.015 PMC5111366

[7] Walker C, Herranz-Martin S, Karyka E, Liao C, Lewis K, Elsayed W, et al. C9orf72 expansion disrupts ATM-mediated chromosomal break repair. Nat Neurosci. 2017;20:1225–35. 28714954 10.1038/nn.4604 PMC5578434

[8] Nihei Y, Mori K, Werner G, Arzberger T, Zhou Q, Khosravi B, et al. Poly-glycine-alanine exacerbates C9orf72 repeat expansion-mediated DNA damage via sequestration of phosphorylated ATM and loss of nuclear hnRNPA3. Acta Neuropathol. 2020;139:99–118. 31642962 10.1007/s00401-019-02082-0 PMC6942035

[9] Yang F, Mahaman YAR, Zhang B, Wang JZ, Liu R, Liu F, et al. C9orf72 poly-PR helps p53 escape from the ubiquitin-proteasome system and promotes its stability. J Neurochem. 2023;166:389–402. 37319115 10.1111/jnc.15872

[10] Wang H, Guo M, Wei H, Chen Y. Targeting p53 pathways: mechanisms, structures, and advances in therapy. Signal Transduct Target Ther. 2023;8:92. 36859359 10.1038/s41392-023-01347-1 PMC9977964

[11] Lucchesi C, Zhang J, Chen X. Modulation of the p53 family network by RNA-binding proteins. Transl Cancer Res. 2016;5:676–84. 28794999 10.21037/tcr.2016.08.30 PMC5546219

[12] Haronikova L, Olivares-Illana V, Wang L, Karakostis K, Chen S, Fahraeus R. The p53 mRNA: an integral part of the cellular stress response. Nucleic Acids Res. 2019;47:3257–71. 30828720 10.1093/nar/gkz124 PMC6468297

[13] Park E, Maquat LE. Staufen-mediated mRNA decay. Wiley Interdiscip Rev RNA. 2013;4:423–35. 23681777 10.1002/wrna.1168 PMC3711692

[14] Heraud-Farlow JE, Kiebler MA. The multifunctional Staufen proteins: conserved roles from neurogenesis to synaptic plasticity. Trends Neurosci. 2014;37:470–9. 25012293 10.1016/j.tins.2014.05.009 PMC4156307

[15] Almasi S, Jasmin BJ. The multifunctional RNA-binding protein Staufen1: an emerging regulator of oncogenesis through its various roles in key cellular events. Cell Mol Life Sci. 2021;78:7145–60. 34633481 10.1007/s00018-021-03965-w PMC8629789

[16] Bonnet-Magnaval F, DesGroseillers L. The Staufen1-dependent cell cycle regulon or how a misregulated RNA-binding protein leads to cancer. Biol Rev Camb Philos Soc. 2021;96:2192–208. 34018319 10.1111/brv.12749

[17] Paul S, Dansithong W, Figueroa KP, Gandelman M, Scoles DR, Pulst SM. Staufen1 in human neurodegeneration. Ann Neurol. 2021;89:1114–28. 33745139 10.1002/ana.26069 PMC9724591

[18] Gandelman M, Dansithong W, Figueroa KP, Paul S, Scoles DR, Pulst SM. Staufen 1 amplifies proapoptotic activation of the unfolded protein response. Cell Death Differ. 2020;27:2942–51. 32415281 10.1038/s41418-020-0553-9 PMC7492261

[19] Paul S, Dansithong W, Gandelman M, Figueroa KP, Zu T, Ranum LPW, Scoles DR, Pulst SM. Staufen Impairs Autophagy in Neurodegeneration. Ann Neurol. 2023;93:398–416. 36151701 10.1002/ana.26515 PMC9892312

[20] Paul S, Dansithong W, Figueroa KP, Scoles DR, Pulst SM. Staufen1 links RNA stress granules and autophagy in a model of neurodegeneration. Nat Commun. 2018;9:3648. 30194296 10.1038/s41467-018-06041-3 PMC6128856

[21] Liu J, Zhang KS, Hu B, Li SG, Li Q, Luo YP, et al. Systematic analysis of RNA regulatory network in rat brain after ischemic stroke. Biomed Res Int. 2018;2018:8354350. 29516010 10.1155/2018/8354350 PMC5817225

[22] Bonnet-Magnaval F, Philippe C, Van Den Berghe L, Prats H, Touriol C, Lacazette E. Hypoxia and ER stress promote Staufen1 expression through an alternative translation mechanism. Biochem Biophys Res Commun. 2016;479:365–71. 27644878 10.1016/j.bbrc.2016.09.082

[23] Pulst SM, Scoles DR, Paul S. Effects of STAU1/staufen1 on autophagy in neurodegenerative diseases. Autophagy. 2023;19:2607–8. 36652469 10.1080/15548627.2023.2169306 PMC10392743

[24] Li CL, Zhou GF, Xie XY, Wang L, Chen X, Pan QL, et al. STAU1 exhibits a dual function by promoting amyloidogenesis and tau phosphorylation in cultured cells. Exp Neurol. 2024;377:114805. 38729552 10.1016/j.expneurol.2024.114805

[25] Zhao R, Huang S, Li J, Gu A, Fu M, Hua W. Excessive STAU1 condensate drives mTOR translation and autophagy dysfunction in neurodegeneration. J Cell Biol. 2024;223:e202311127 38913026 10.1083/jcb.202311127 PMC11194678

[26] Liberzon A, Birger C, Thorvaldsdottir H, Ghandi M, Mesirov JP, Tamayo P. The Molecular Signatures Database (MSigDB) hallmark gene set collection. Cell Syst. 2015;1:417–25. 26771021 10.1016/j.cels.2015.12.004 PMC4707969

[27] Kramer A, Green J, Pollard J Jr, Tugendreich S. Causal analysis approaches in Ingenuity Pathway Analysis. Bioinformatics. 2014;30:523–30. 24336805 10.1093/bioinformatics/btt703 PMC3928520

[28] Khan H, Bangar A, Grewal AK, Bansal P, Singh TG. Caspase-mediated regulation of the distinct signaling pathways and mechanisms in neuronal survival. Int Immunopharmacol. 2022;110:108951. 35717837 10.1016/j.intimp.2022.108951

[29] Yadav P, Yadav R, Jain S, Vaidya A. Caspase-3: a primary target for natural and synthetic compounds for cancer therapy. Chem Biol Drug Des. 2021;98:144–65. 33963665 10.1111/cbdd.13860

[30] Chow KC, Ross WE. Topoisomerase-specific drug sensitivity in relation to cell cycle progression. Mol Cell Biol. 1987;7:3119–23. 2823120 10.1128/mcb.7.9.3119-3123.1987 PMC367945

[31] Vassilev LT, Vu BT, Graves B, Carvajal D, Podlaski F, Filipovic Z, et al. In vivo activation of the p53 pathway by small-molecule antagonists of MDM2. Science. 2004;303:844–8. 14704432 10.1126/science.1092472

[32] Verma R, Rigatti MJ, Belinsky GS, Godman CA, Giardina C. DNA damage response to the Mdm2 inhibitor nutlin-3. Biochem Pharm. 2010;79:565–74. 19788889 10.1016/j.bcp.2009.09.020 PMC2794967

[33] Vessey JP, Macchi P, Stein JM, Mikl M, Hawker KN, Vogelsang P, et al. A loss of function allele for murine Staufen1 leads to impairment of dendritic Staufen1-RNP delivery and dendritic spine morphogenesis. Proc Natl Acad Sci USA. 2008;105:16374–9. 18922781 10.1073/pnas.0804583105 PMC2567905

[34] Zhang Y, Pak C, Han Y, Ahlenius H, Zhang Z, Chanda S, et al. Rapid single-step induction of functional neurons from human pluripotent stem cells. Neuron. 2013;78:785–98. 23764284 10.1016/j.neuron.2013.05.029 PMC3751803

[35] Wang C, Ward ME, Chen R, Liu K, Tracy TE, Chen X, et al. Scalable production of iPSC-derived human neurons to identify tau-lowering compounds by high-content screening. Stem Cell Rep. 2017;9:1221–33. 10.1016/j.stemcr.2017.08.019 PMC5639430 28966121

[36] Fernandopulle MS, Prestil R, Grunseich C, Wang C, Gan L, Ward ME. Transcription factor-mediated differentiation of human iPSCs into neurons. Curr Protoc Cell Biol. 2018;79:e51. 29924488 10.1002/cpcb.51 PMC6993937

[37] Abbas T, Dutta A. p21 in cancer: intricate networks and multiple activities. Nat Rev Cancer. 2009;9:400–14. 19440234 10.1038/nrc2657 PMC2722839

[38] Al Bitar S, Gali-Muhtasib H. The role of the cyclin dependent kinase inhibitor p21(cip1/waf1) in targeting cancer: molecular mechanisms and novel therapeutics. Cancers. 2019;11:1475 31575057 10.3390/cancers11101475 PMC6826572

[39] Hafner A, Bulyk ML, Jambhekar A, Lahav G. The multiple mechanisms that regulate p53 activity and cell fate. Nat Rev Mol Cell Biol. 2019;20:199–210. 30824861 10.1038/s41580-019-0110-x

[40] Maor-Nof M, Shipony Z, Lopez-Gonzalez R, Nakayama L, Zhang YJ, Couthouis J, et al. p53 is a central regulator driving neurodegeneration caused by C9orf72 poly(PR). Cell. 2021;184:689–708.e20. 33482083 10.1016/j.cell.2020.12.025 PMC7886018

[41] Lopez-Gonzalez R, Yang D, Pribadi M, Kim TS, Krishnan G, Choi SY, et al. Partial inhibition of the overactivated Ku80-dependent DNA repair pathway rescues neurodegeneration in C9ORF72-ALS/FTD. Proc Natl Acad Sci USA. 2019;116:9628–33. 31019093 10.1073/pnas.1901313116 PMC6511021

[42] Wang R, Xu X, Hao Z, Zhang S, Wu D, Sun H, et al. Poly-PR in C9ORF72-Related amyotrophic lateral sclerosis/frontotemporal dementia causes neurotoxicity by clathrin-dependent endocytosis. Neurosci Bull. 2019;35:889–900. 31148094 10.1007/s12264-019-00395-4 PMC6754483

[43] Liu Y, Pattamatta A, Zu T, Reid T, Bardhi O, Borchelt DR, et al. C9orf72 BAC Mouse model with motor deficits and neurodegenerative features of ALS/FTD. Neuron. 2016;90:521–34. 27112499 10.1016/j.neuron.2016.04.005

[44] de Morree A, van Velthoven CTJ, Gan Q, Salvi JS, Klein JDD, Akimenko I, et al. Staufen1 inhibits MyoD translation to actively maintain muscle stem cell quiescence. Proc Natl Acad Sci USA. 2017;114:E8996–E9005. 29073096 10.1073/pnas.1708725114 PMC5664522

[45] Porter JR, Fisher BE, Baranello L, Liu JC, Kambach DM, Nie Z, et al. Global inhibition with specific activation: how p53 and MYC redistribute the transcriptome in the DNA double-strand break response. Mol Cell. 2017;67:1013–25.e9. 28867293 10.1016/j.molcel.2017.07.028 PMC5657607

[46] Ravel-Chapuis A, Crawford TE, Blais-Crepeau ML, Belanger G, Richer CT, Jasmin BJ. The RNA-binding protein Staufen1 impairs myogenic differentiation via a c-myc-dependent mechanism. Mol Biol Cell. 2014;25:3765–78. 25208565 10.1091/mbc.E14-04-0895 PMC4230783

[47] Weidensdorfer D, Stohr N, Baude A, Lederer M, Kohn M, Schierhorn A, et al. Control of c-myc mRNA stability by IGF2BP1-associated cytoplasmic RNPs. RNA. 2009;15:104–15. 19029303 10.1261/rna.1175909 PMC2612774

[48] Scully R, Xie A. Double strand break repair functions of histone H2AX. Mutat Res. 2013;750:5–14. 23916969 10.1016/j.mrfmmm.2013.07.007 PMC3818383

[49] Rogakou EP, Pilch DR, Orr AH, Ivanova VS, Bonner WM. DNA double-stranded breaks induce histone H2AX phosphorylation on serine 139. J Biol Chem. 1998;273:5858–68. 9488723 10.1074/jbc.273.10.5858

[50] Laver JD, Li X, Ancevicius K, Westwood JT, Smibert CA, Morris QD, et al. Genome-wide analysis of Staufen-associated mRNAs identifies secondary structures that confer target specificity. Nucleic Acids Res. 2013;41:9438–60. 23945942 10.1093/nar/gkt702 PMC3814352

[51] Dugre-Brisson S, Elvira G, Boulay K, Chatel-Chaix L, Mouland AJ, DesGroseillers L. Interaction of Staufen1 with the 5’ end of mRNA facilitates translation of these RNAs. Nucleic Acids Res. 2005;33:4797–812. 16126845 10.1093/nar/gki794 PMC1193567

[52] Elbarbary RA, Li W, Tian B, Maquat LE. STAU1 binding 3’ UTR IRAlus complements nuclear retention to protect cells from PKR-mediated translational shutdown. Genes Dev. 2013;27:1495–510. 23824540 10.1101/gad.220962.113 PMC3713430

[53] Gong C, Maquat LE. lncRNAs transactivate STAU1-mediated mRNA decay by duplexing with 3’ UTRs via Alu elements. Nature. 2011;470:284–8. 21307942 10.1038/nature09701 PMC3073508

[54] Kim YK, Furic L, Desgroseillers L, Maquat LE. Mammalian Staufen1 recruits Upf1 to specific mRNA 3’UTRs so as to elicit mRNA decay. Cell. 2005;120:195–208. 15680326 10.1016/j.cell.2004.11.050

[55] Gowravaram M, Schwarz J, Khilji SK, Urlaub H, Chakrabarti S. Insights into the assembly and architecture of a Staufen-mediated mRNA decay (SMD)-competent mRNP. Nat Commun. 2019;10:5054. 31699982 10.1038/s41467-019-13080-x PMC6838198

[56] Fielder E, von Zglinicki T, Jurk D. The DNA damage response in neurons: die by apoptosis or survive in a senescence-like state?. J Alzheimers Dis. 2017;60:S107–S31. 28436392 10.3233/JAD-161221

[57] Ghram M, Bonnet-Magnaval F, Hotea DI, Doran B, Ly S, DesGroseillers L. Staufen1 is essential for cell-cycle transitions and cell proliferation via the control of E2F1 expression. J Mol Biol. 2020;432:3881–97. 32335035 10.1016/j.jmb.2020.04.016

[58] Boulay K, Ghram M, Viranaicken W, Trepanier V, Mollet S, Frechina C, et al. Cell cycle-dependent regulation of the RNA-binding protein Staufen1. Nucleic Acids Res. 2014;42:7867–83. 24906885 10.1093/nar/gku506 PMC4081104

[59] Ravel-Chapuis A, Belanger G, Yadava RS, Mahadevan MS, DesGroseillers L, Cote J, et al. The RNA-binding protein Staufen1 is increased in DM1 skeletal muscle and promotes alternative pre-mRNA splicing. J Cell Biol. 2012;196:699–712. 22431750 10.1083/jcb.201108113 PMC3308689

[60] Konopka A, Atkin JD. DNA damage, defective DNA repair, and neurodegeneration in amyotrophic lateral sclerosis. Front Aging Neurosci. 2022;14:786420. 35572138 10.3389/fnagi.2022.786420 PMC9093740

[61] Wang H, Kodavati M, Britz GW, Hegde ML. DNA damage and repair deficiency in ALS/FTD-associated neurodegeneration: from molecular mechanisms to therapeutic implication. Front Mol Neurosci. 2021;14:784361. 34975400 10.3389/fnmol.2021.784361 PMC8716463

[62] Martin LJ. p53 is abnormally elevated and active in the CNS of patients with amyotrophic lateral sclerosis. Neurobiol Dis. 2000;7:613–22. 11114260 10.1006/nbdi.2000.0314

[63] Sun Y, Curle AJ, Haider AM, Balmus G. The role of DNA damage response in amyotrophic lateral sclerosis. Essays Biochem. 2020;64:847–61. 33078197 10.1042/EBC20200002 PMC7588667

[64] Mitra J, Guerrero EN, Hegde PM, Liachko NF, Wang H, Vasquez V, et al. Motor neuron disease-associated loss of nuclear TDP-43 is linked to DNA double-strand break repair defects. Proc Natl Acad Sci USA. 2019;116:4696–705. 30770445 10.1073/pnas.1818415116 PMC6410842

[65] Fitzmaurice PS, Shaw IC, Kleiner HE, Miller RT, Monks TJ, Lau SS, et al. Evidence for DNA damage in amyotrophic lateral sclerosis. Muscle Nerve. 1996;19:797–8. 8609941

[66] Shaikh AY, Martin LJ. DNA base-excision repair enzyme apurinic/apyrimidinic endonuclease/redox factor-1 is increased and competent in the brain and spinal cord of individuals with amyotrophic lateral sclerosis. Neuromolecular Med. 2002;2:47–60. 12230304 10.1007/s12017-002-0038-7

[67] Hill SJ, Mordes DA, Cameron LA, Neuberg DS, Landini S, Eggan K, et al. Two familial ALS proteins function in prevention/repair of transcription-associated DNA damage. Proc Natl Acad Sci USA. 2016;113:E7701–E9. 27849576 10.1073/pnas.1611673113 PMC5137757

[68] Liu EY, Russ J, Cali CP, Phan JM, Amlie-Wolf A, Lee EB. Loss of nuclear TDP-43 is associated with decondensation of LINE retrotransposons. Cell Rep. 2019;27:1409–21.e6. 31042469 10.1016/j.celrep.2019.04.003 PMC6508629

[69] Lee K, Suzuki H, Aiso S, Matsuoka M. Overexpression of TDP-43 causes partially p53-dependent G2/M arrest and p53-independent cell death in HeLa cells. Neurosci Lett. 2012;506:271–6. 22133803 10.1016/j.neulet.2011.11.021

[70] Gandelman M, Levy M, Cassina P, Barbeito L, Beckman JS. P2X7 receptor-induced death of motor neurons by a peroxynitrite/FAS-dependent pathway. J Neurochem. 2013;126:382–8. 23646980 10.1111/jnc.12286 PMC3716845

[71] Gandelman M, Peluffo H, Beckman JS, Cassina P, Barbeito L. Extracellular ATP and the P2X7 receptor in astrocyte-mediated motor neuron death: implications for amyotrophic lateral sclerosis. J Neuroinflamm. 2010;7:33. 10.1186/1742-2094-7-33 PMC2901222 20534165

[72] Pantazis CB, Yang A, Lara E, McDonough JA, Blauwendraat C, Peng L, et al. A reference human induced pluripotent stem cell line for large-scale collaborative studies. Cell Stem Cell. 2022;29:1685–702.e22. 36459969 10.1016/j.stem.2022.11.004 PMC9782786

[73] Challis RC, Ravindra Kumar S, Chan KY, Challis C, Beadle K, Jang MJ, et al. Systemic AAV vectors for widespread and targeted gene delivery in rodents. Nat Protoc. 2019;14:379–414. 30626963 10.1038/s41596-018-0097-3 PMC13333184

[74] DeJesus-Hernandez M, Mackenzie IR, Boeve BF, Boxer AL, Baker M, Rutherford NJ, et al. Expanded GGGGCC hexanucleotide repeat in noncoding region of C9ORF72 causes chromosome 9p-linked FTD and ALS. Neuron. 2011;72:245–56. 21944778 10.1016/j.neuron.2011.09.011 PMC3202986

[75] Dobin A, Davis CA, Schlesinger F, Drenkow J, Zaleski C, Jha S, et al. STAR: ultrafast universal RNA-seq aligner. Bioinformatics. 2013;29:15–21. 23104886 10.1093/bioinformatics/bts635 PMC3530905

[76] Bushnell B, Rood J, Singer E. BBMerge - Accurate paired shotgun read merging via overlap. PLoS One. 2017;12:e0185056. 29073143 10.1371/journal.pone.0185056 PMC5657622

[77] Martin M. Cutadapt removes adapter sequences from high-throughput sequencing reads. EMBnet j. 2011;17:3.

[78] Liao Y, Smyth GK, Shi W. featureCounts: an efficient general purpose program for assigning sequence reads to genomic features. Bioinformatics. 2014;30:923–30. 24227677 10.1093/bioinformatics/btt656

[79] Ewels P, Magnusson M, Lundin S, Kaller M. MultiQC: summarize analysis results for multiple tools and samples in a single report. Bioinformatics. 2016;32:3047–8. 27312411 10.1093/bioinformatics/btw354 PMC5039924

[80] Love MI, Huber W, Anders S. Moderated estimation of fold change and dispersion for RNA-seq data with DESeq2. Genome Biol. 2014;15:550. 25516281 10.1186/s13059-014-0550-8 PMC4302049

[81] Korotkevich G, Sukhov V, Budin N, Shpak B, Artyomov MN, Sergushichev A Fast gene set enrichment analysis. bioRxiv. 2021. 10.1101/060012.

[82] Gandelman M, Dansithong W, Kales SC, Paul S, Maag G, Aoyama E, et al. The AKT modulator A-443654 reduces alpha-synuclein expression and normalizes ER stress and autophagy. J Biol Chem. 2021;297:101191. 34520759 10.1016/j.jbc.2021.101191 PMC8482485

[83] Gyori BM, Venkatachalam G, Thiagarajan PS, Hsu D, Clement MV. OpenComet: an automated tool for comet assay image analysis. Redox Biol. 2014;2:457–65. 24624335 10.1016/j.redox.2013.12.020 PMC3949099

