# Supplementary material for: Targeting STAU1 prevents p53 apoptotic signaling in neurodegeneration
Source: Cell Death Dis. 2025 Oct 27;16(1):761. doi: 10.1038/s41419-025-08067-0 (PMC12559333; doi:10.1038/s41419-025-08067-0)
Supplement: Supplementary file 1 — Supplemental material [file 41419_2025_8067_MOESM1_ESM.docx]

**Targeting STAU1 prevents p53 apoptotic signaling and DNA damage in neurodegeneration.**

Mandi Gandelman, Sharan Paul, Karla P Figueroa, Justine Sundrud, Warunee Dansithong, Daniel R Scoles, Stefan M Pulst.

**SUPPLEMENTARY INFORMATION**


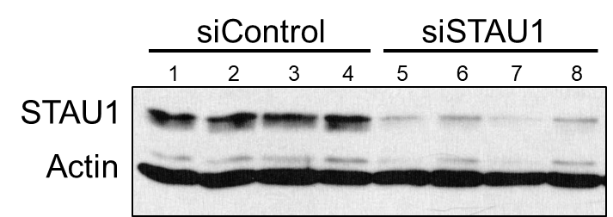


SI Fig.1. STAU1 Knockdown in HEK293 cells. Western blot of lysates from HEK293 cells treated with siSTAU1 vs siControl, for the 8 samples utilized for RNAseq.


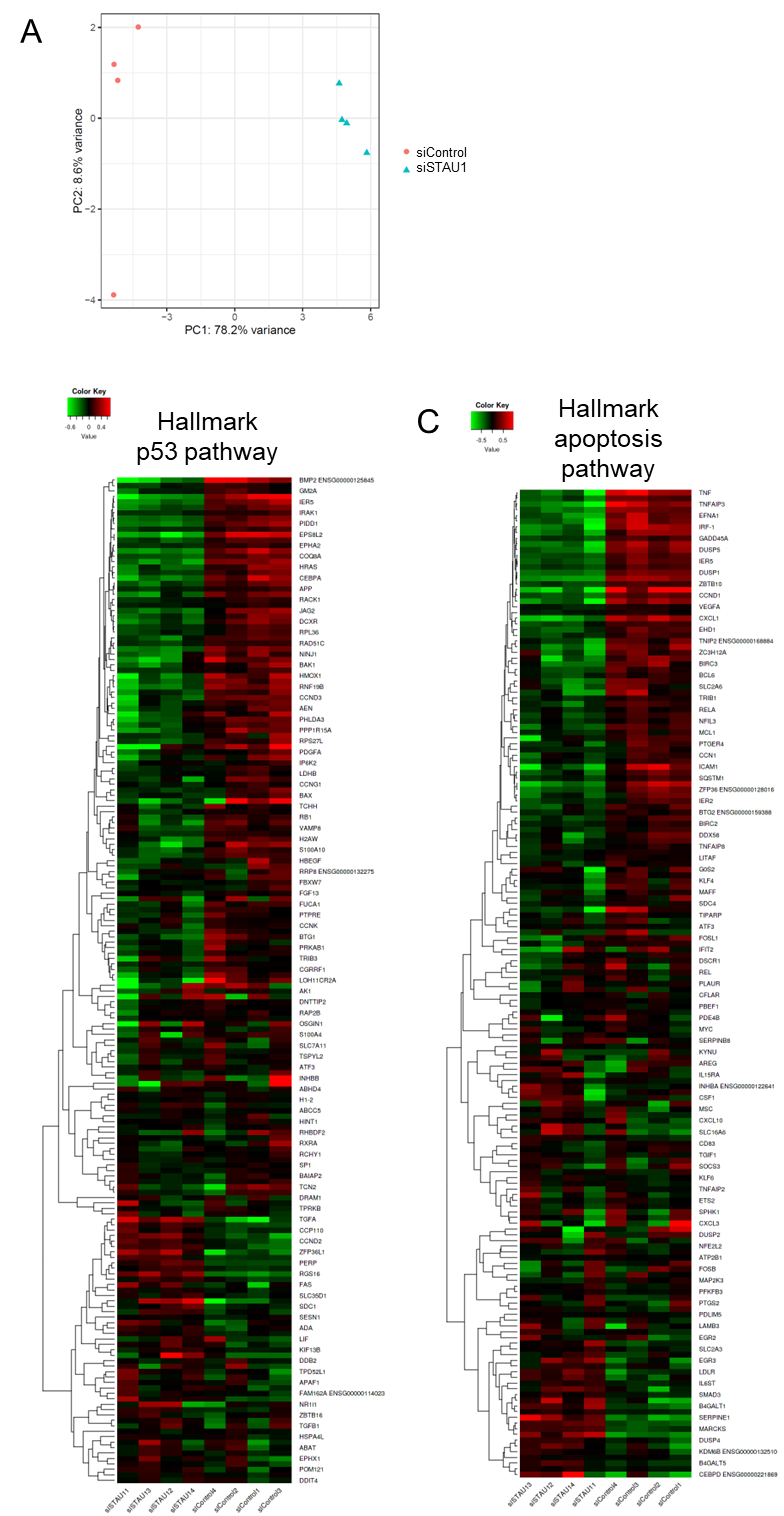


SI Fig. 2. (A) PCA chart of siControl and siSTAU1 samples. (B, C) Heatmap of Hallmark pathways. (B) p53 and (C) apoptosis pathways.


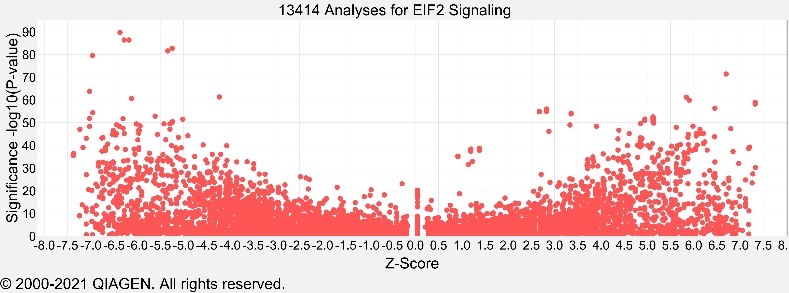

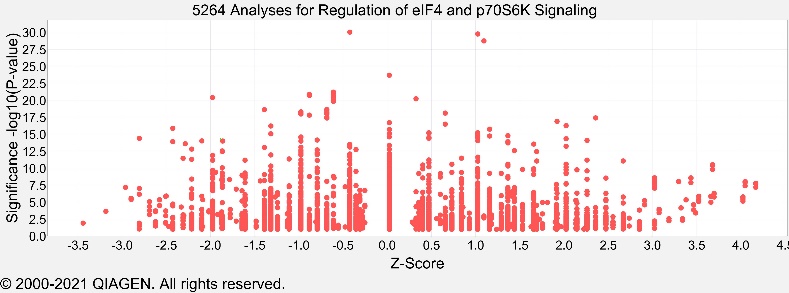

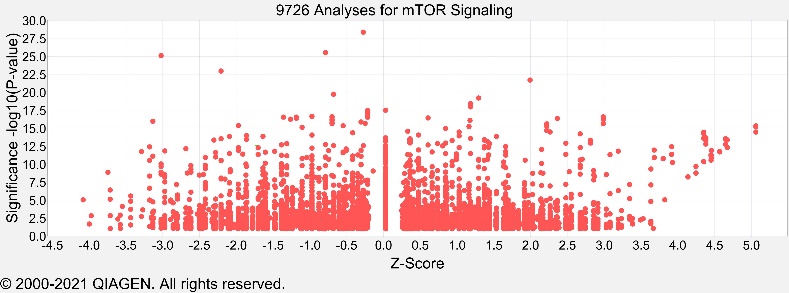


SI Fig. 3. IPA activity plots for canonical pathways. Activity plots for eIF2 signaling, eIF4 and p70S6K signaling and mTOR signaling pathways. The green circle indicates the siSTAU1 dataset.


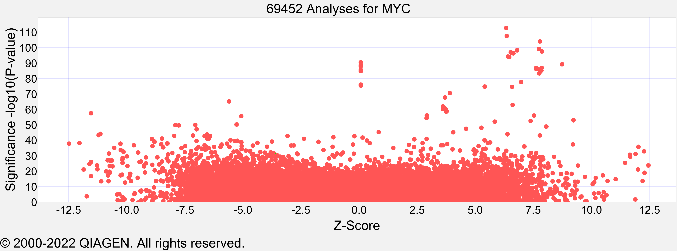

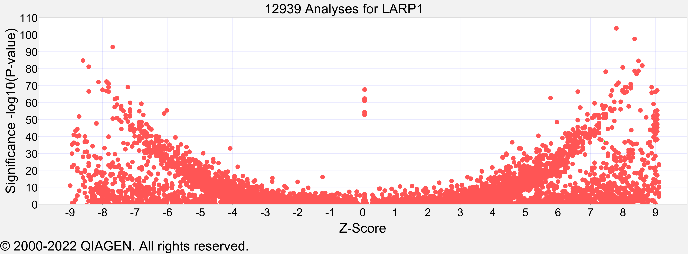

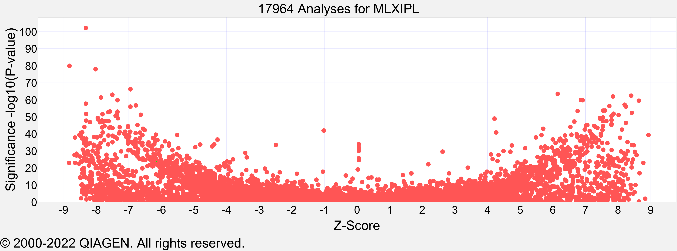

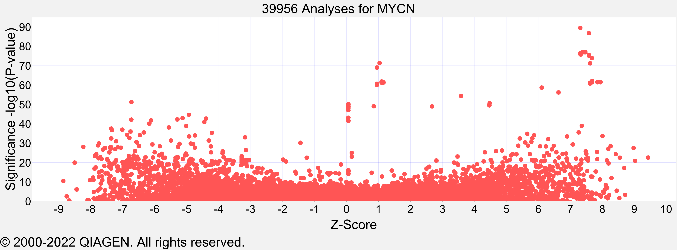


SI Fig. 4. IPA activity plots for selected Upstream Regulators. The green circle indicates the siSTAU1 dataset


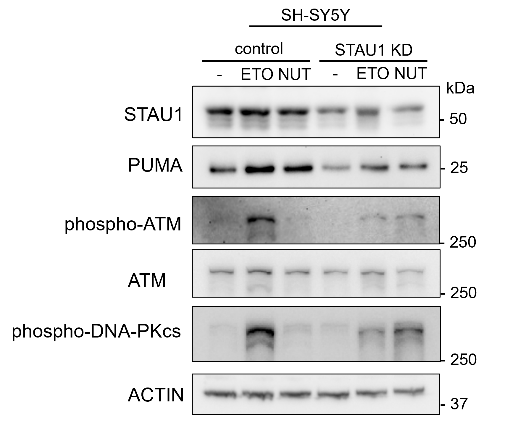


SI Fig. 5. The DDR is active after STAU1 KD. Evaluation of the DDR in SH-SY5Y cells by western blot of phospho-ATM and phosphor-DNA-PKcs.


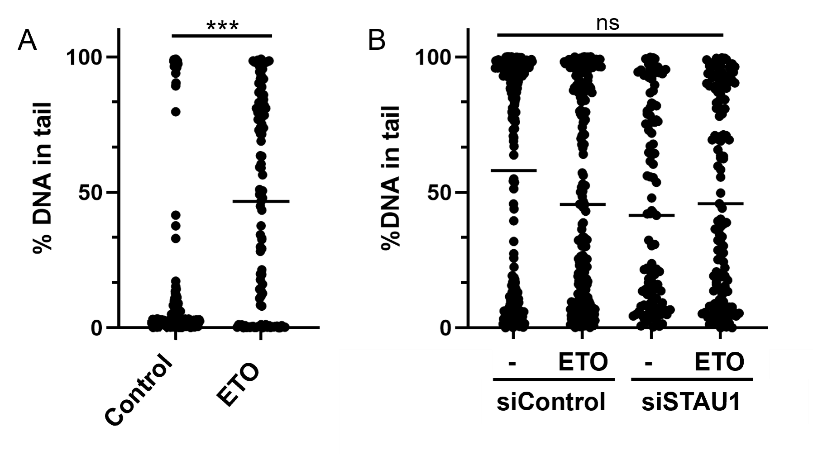


SI Fig. 6. Evaluation of DNA damage with a Comet assay in iNeurons. (A) As a positive control, iNeurons were treated with 10 µM etoposide for 1 hour. (B) iNeurons transfected with siControl or siSTAU1 displayed a high baseline of DNA damage, and no further increases were elicited by 10 µM Etoposide for 1 hour, suggesting the high sensitivity and low dynamic range of the Comet assay is not appropriate for our experimental conditions. Each dot in the graph represents an analyzed cell and *** is p<0.01.


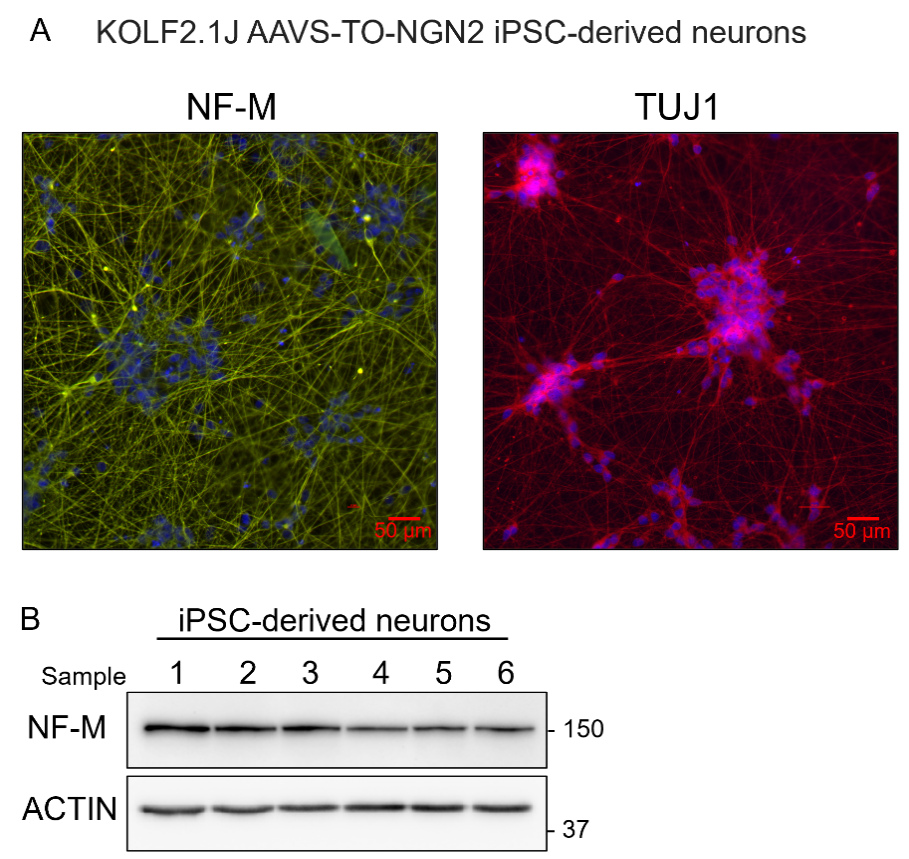


SI Fig. 7. Characterization of induced neurons (iNeurons). **(A)** Immunofluorescence staining of iNeurons showing morphology and expression of neuronal markers NF-M and TUJ1 **(B)** Western blot of iNeurons showing expression of NF-M.

Supplemental Table 1. Significant (-log10(p-value)>1.3) IPA canonical pathways.

| © 2000-2021 QIAGEN. All rights reserved. | |  |  |
| --- | --- | --- | --- |
| **Ingenuity Canonical Pathways** | **-log10(p-value)** | **Ratio** | **z-score** |
| EIF2 Signaling | 31.3 | 0.442 | -5.859 |
| Regulation of eIF4 and p70S6K Signaling | 14.3 | 0.352 | -1.886 |
| mTOR Signaling | 12.6 | 0.316 | -1.732 |
| Coronavirus Pathogenesis Pathway | 10.5 | 0.3 | 2.213 |
| Molecular Mechanisms of Cancer | 9.32 | 0.23 | #NUM! |
| Senescence Pathway | 8.4 | 0.249 | -0.5 |
| Cyclins and Cell Cycle Regulation | 7.96 | 0.369 | 0 |
| Caveolar-mediated Endocytosis Signaling | 7.38 | 0.373 | #NUM! |
| Integrin Signaling | 7.38 | 0.263 | -0.14 |
| HER-2 Signaling in Breast Cancer | 7.15 | 0.256 | -1.286 |
| Glioma Signaling | 7.05 | 0.306 | 0.688 |
| Protein Ubiquitination Pathway | 6.91 | 0.24 | #NUM! |
| Axonal Guidance Signaling | 6.85 | 0.207 | #NUM! |
| Pulmonary Fibrosis Idiopathic Signaling Pathway | 6.62 | 0.227 | 1.522 |
| BEX2 Signaling Pathway | 6.56 | 0.346 | 0.378 |
| ILK Signaling | 6.49 | 0.258 | -0.905 |
| Cell Cycle Control of Chromosomal Replication | 6.39 | 0.393 | 1.706 |
| Epithelial Adherens Junction Signaling | 6.35 | 0.274 | 1.372 |
| IL-8 Signaling | 6.34 | 0.251 | -1.64 |
| Actin Cytoskeleton Signaling | 5.95 | 0.237 | 1.021 |
| Cell Cycle: G1/S Checkpoint Regulation | 5.91 | 0.353 | 0.894 |
| Pancreatic Adenocarcinoma Signaling | 5.9 | 0.286 | -1.043 |
| Ovarian Cancer Signaling | 5.84 | 0.266 | -1.147 |
| Phagosome Maturation | 5.77 | 0.264 | #NUM! |
| Regulation of Cellular Mechanics by Calpain Protease | 5.63 | 0.315 | -1.291 |
| CDK5 Signaling | 5.6 | 0.289 | -0.186 |
| Semaphorin Signaling in Neurons | 5.51 | 0.355 | #NUM! |
| Small Cell Lung Cancer Signaling | 5.41 | 0.302 | 0 |
| Synaptogenesis Signaling Pathway | 5.38 | 0.217 | 2 |
| Neuregulin Signaling | 5.33 | 0.282 | -0.853 |
| PI3K/AKT Signaling | 5.28 | 0.241 | -0.667 |
| Hepatic Fibrosis / Hepatic Stellate Cell Activation | 5.24 | 0.242 | #NUM! |
| PTEN Signaling | 5.23 | 0.26 | 0.174 |
| Hepatic Fibrosis Signaling Pathway | 5.08 | 0.2 | -1.195 |
| Wound Healing Signaling Pathway | 4.89 | 0.222 | 0.267 |
| Prostate Cancer Signaling | 4.87 | 0.277 | #NUM! |
| ERK/MAPK Signaling | 4.72 | 0.229 | -1.718 |
| Production of Nitric Oxide and Reactive Oxygen Species in Macrophages | 4.71 | 0.236 | -3.162 |
| Hereditary Breast Cancer Signaling | 4.62 | 0.254 | #NUM! |
| Protein Kinase A Signaling | 4.55 | 0.197 | -0.739 |
| Thrombin Signaling | 4.5 | 0.226 | 0.164 |
| Chronic Myeloid Leukemia Signaling | 4.41 | 0.271 | #NUM! |
| HIF1α Signaling | 4.4 | 0.226 | 0.905 |
| ATM Signaling | 4.37 | 0.278 | 0 |
| Actin Nucleation by ARP-WASP Complex | 4.27 | 0.28 | -1.414 |
| Aryl Hydrocarbon Receptor Signaling | 4.23 | 0.239 | -0.6 |
| VEGF Signaling | 4.2 | 0.273 | -1.606 |
| Non-Small Cell Lung Cancer Signaling | 4.18 | 0.277 | -0.535 |
| RAN Signaling | 4.14 | 0.529 | 1 |
| RAC Signaling | 4.13 | 0.246 | -0.192 |
| Bladder Cancer Signaling | 4.13 | 0.259 | -2.121 |
| Tight Junction Signaling | 4.12 | 0.23 | #NUM! |
| Inhibition of ARE-Mediated mRNA Degradation Pathway | 4.1 | 0.236 | 0.557 |
| Signaling by Rho Family GTPases | 4.1 | 0.209 | 0.302 |
| Agrin Interactions at Neuromuscular Junction | 4.04 | 0.3 | -0.943 |
| Semaphorin Neuronal Repulsive Signaling Pathway | 4.02 | 0.238 | 0.522 |
| Insulin Secretion Signaling Pathway | 4.01 | 0.207 | -0.14 |
| Role of MAPK Signaling in Promoting the Pathogenesis of Influenza | 3.95 | 0.257 | -2.646 |
| Cholecystokinin/Gastrin-mediated Signaling | 3.91 | 0.252 | -0.784 |
| Oxytocin Signaling Pathway | 3.88 | 0.204 | 1.604 |
| Sumoylation Pathway | 3.88 | 0.262 | 0.229 |
| Sirtuin Signaling Pathway | 3.86 | 0.202 | -1.155 |
| Glioblastoma Multiforme Signaling | 3.85 | 0.228 | -0.73 |
| Autophagy | 3.83 | 0.216 | -1.64 |
| Ephrin Receptor Signaling | 3.83 | 0.219 | -0.2 |
| GP6 Signaling Pathway | 3.74 | 0.244 | 3.286 |
| RHOGDI Signaling | 3.73 | 0.214 | -1.372 |
| Kinetochore Metaphase Signaling Pathway | 3.69 | 0.252 | 0 |
| FAK Signaling | 3.66 | 0.248 | #NUM! |
| Ceramide Signaling | 3.64 | 0.267 | -1.964 |
| Insulin Receptor Signaling | 3.64 | 0.236 | -0.365 |
| Coronavirus Replication Pathway | 3.6 | 0.333 | 2.84 |
| Role of BRCA1 in DNA Damage Response | 3.59 | 0.275 | 1.732 |
| NGF Signaling | 3.59 | 0.246 | -2.6 |
| Sertoli Cell-Sertoli Cell Junction Signaling | 3.58 | 0.214 | #NUM! |
| Germ Cell-Sertoli Cell Junction Signaling | 3.53 | 0.222 | #NUM! |
| GADD45 Signaling | 3.46 | 0.45 | #NUM! |
| Cell Cycle Regulation by BTG Family Proteins | 3.45 | 0.351 | -1.633 |
| Reelin Signaling in Neurons | 3.44 | 0.238 | 1.095 |
| Apoptosis Signaling | 3.41 | 0.25 | 0.6 |
| Regulation of Actin-based Motility by Rho | 3.35 | 0.241 | -0.426 |
| Glioma Invasiveness Signaling | 3.3 | 0.274 | -1 |
| Huntington's Disease Signaling | 3.28 | 0.196 | -0.192 |
| Gαq Signaling | 3.27 | 0.218 | -0.186 |
| Type II Diabetes Mellitus Signaling | 3.22 | 0.222 | -0.728 |
| Hypoxia Signaling in the Cardiovascular System | 3.21 | 0.27 | -2.121 |
| PI3K Signaling in B Lymphocytes | 3.12 | 0.224 | -0.378 |
| Cell Cycle: G2/M DNA Damage Checkpoint Regulation | 3.05 | 0.3 | -0.632 |
| IGF-1 Signaling | 3.03 | 0.24 | -1.886 |
| Iron homeostasis signaling pathway | 3.02 | 0.223 | #NUM! |
| HOTAIR Regulatory Pathway | 3.02 | 0.215 | 2.263 |
| FAT10 Signaling Pathway | 2.96 | 0.286 | 0 |
| dTMP De Novo Biosynthesis | 2.95 | 0.8 | 1 |
| Pyrimidine Deoxyribonucleotides De Novo Biosynthesis I | 2.92 | 0.391 | -0.333 |
| PEDF Signaling | 2.87 | 0.25 | -2.357 |
| iNOS Signaling | 2.85 | 0.298 | -2.53 |
| Ephrin A Signaling | 2.85 | 0.298 | #NUM! |
| Death Receptor Signaling | 2.82 | 0.24 | 0.426 |
| Regulation Of The Epithelial Mesenchymal Transition By Growth Factors Pathway | 2.81 | 0.203 | -0.822 |
| Paxillin Signaling | 2.79 | 0.231 | -0.688 |
| Pulmonary Healing Signaling Pathway | 2.77 | 0.201 | -0.316 |
| NRF2-mediated Oxidative Stress Response | 2.77 | 0.194 | -3.153 |
| HGF Signaling | 2.76 | 0.22 | -2.294 |
| MSP-RON Signaling In Cancer Cells Pathway | 2.76 | 0.217 | -0.73 |
| p53 Signaling | 2.69 | 0.235 | 0.258 |
| Virus Entry via Endocytic Pathways | 2.68 | 0.231 | #NUM! |
| BMP signaling pathway | 2.66 | 0.241 | -1.414 |
| Estrogen Receptor Signaling | 2.63 | 0.175 | -0.405 |
| GNRH Signaling | 2.63 | 0.2 | -0.378 |
| Folate Transformations I | 2.6 | 0.556 | 0.447 |
| EGF Signaling | 2.59 | 0.273 | -0.535 |
| 3-phosphoinositide Degradation | 2.59 | 0.199 | 0.365 |
| Dilated Cardiomyopathy Signaling Pathway | 2.56 | 0.209 | 0.218 |
| ERK5 Signaling | 2.54 | 0.25 | 0.943 |
| CSDE1 Signaling Pathway | 2.51 | 0.268 | 0.258 |
| fMLP Signaling in Neutrophils | 2.5 | 0.214 | 0 |
| Estrogen-mediated S-phase Entry | 2.49 | 0.346 | -0.707 |
| Unfolded protein response | 2.47 | 0.233 | -0.632 |
| Ferroptosis Signaling Pathway | 2.45 | 0.212 | -0.577 |
| Mechanisms of Viral Exit from Host Cells | 2.45 | 0.293 | #NUM! |
| Remodeling of Epithelial Adherens Junctions | 2.43 | 0.25 | 0.447 |
| LPS-stimulated MAPK Signaling | 2.43 | 0.235 | -3.441 |
| Regulation of the Epithelial-Mesenchymal Transition Pathway | 2.42 | 0.195 | #NUM! |
| Endocannabinoid Cancer Inhibition Pathway | 2.42 | 0.207 | 0 |
| Acute Myeloid Leukemia Signaling | 2.41 | 0.231 | -1 |
| RANK Signaling in Osteoclasts | 2.41 | 0.231 | -1.414 |
| Cardiac Hypertrophy Signaling | 2.41 | 0.185 | -0.845 |
| Gα12/13 Signaling | 2.4 | 0.211 | -1.961 |
| TNFR1 Signaling | 2.4 | 0.269 | -1.387 |
| 14-3-3-mediated Signaling | 2.4 | 0.213 | -0.894 |
| Amyotrophic Lateral Sclerosis Signaling | 2.39 | 0.217 | -0.943 |
| IL-6 Signaling | 2.35 | 0.211 | -3.138 |
| Salvage Pathways of Pyrimidine Ribonucleotides | 2.35 | 0.224 | -1.091 |
| TNFR2 Signaling | 2.35 | 0.312 | -1.414 |
| D-myo-inositol (1,4,5,6)-Tetrakisphosphate Biosynthesis | 2.3 | 0.196 | 0.784 |
| D-myo-inositol (3,4,5,6)-tetrakisphosphate Biosynthesis | 2.3 | 0.196 | 0.784 |
| Synaptic Long Term Potentiation | 2.3 | 0.209 | -0.6 |
| Xenobiotic Metabolism Signaling | 2.3 | 0.179 | #NUM! |
| GM-CSF Signaling | 2.29 | 0.243 | -1.604 |
| nNOS Signaling in Skeletal Muscle Cells | 2.29 | 0.271 | #NUM! |
| WNT/β-catenin Signaling | 2.29 | 0.197 | 1.134 |
| Colorectal Cancer Metastasis Signaling | 2.27 | 0.181 | -1.761 |
| Opioid Signaling Pathway | 2.26 | 0.18 | 0.745 |
| Clathrin-mediated Endocytosis Signaling | 2.26 | 0.192 | #NUM! |
| Notch Signaling | 2.25 | 0.289 | -0.333 |
| Androgen Signaling | 2.19 | 0.195 | 1.213 |
| Nitric Oxide Signaling in the Cardiovascular System | 2.19 | 0.21 | 0.894 |
| Superpathway of Inositol Phosphate Compounds | 2.19 | 0.184 | 0.169 |
| D-myo-inositol-5-phosphate Metabolism | 2.18 | 0.19 | 0.756 |
| Mitotic Roles of Polo-Like Kinase | 2.18 | 0.242 | 1.134 |
| Role of NFAT in Cardiac Hypertrophy | 2.15 | 0.185 | 0.87 |
| 4-1BB Signaling in T Lymphocytes | 2.14 | 0.294 | -1.89 |
| BAG2 Signaling Pathway | 2.14 | 0.226 | 1.265 |
| Calcium Signaling | 2.13 | 0.185 | 2.117 |
| Mitochondrial Dysfunction | 2.12 | 0.193 | #NUM! |
| HIPPO signaling | 2.08 | 0.224 | -0.302 |
| NER (Nucleotide Excision Repair, Enhanced Pathway) | 2.08 | 0.214 | 2.183 |
| 3-phosphoinositide Biosynthesis | 2.06 | 0.185 | 0.557 |
| Tumor Microenvironment Pathway | 2.06 | 0.19 | -0.174 |
| UVC-Induced MAPK Signaling | 2.05 | 0.255 | -1.732 |
| STAT3 Pathway | 2.03 | 0.2 | -0.209 |
| Adipogenesis pathway | 2.03 | 0.2 | #NUM! |
| PDGF Signaling | 2.03 | 0.221 | -2.524 |
| Leukocyte Extravasation Signaling | 2.02 | 0.187 | -0.354 |
| Granzyme B Signaling | 1.99 | 0.375 | 0.816 |
| Mismatch Repair in Eukaryotes | 1.99 | 0.375 | #NUM! |
| Dopamine-DARPP32 Feedback in cAMP Signaling | 1.98 | 0.188 | 0.756 |
| PAK Signaling | 1.95 | 0.203 | -1.147 |
| Macropinocytosis Signaling | 1.92 | 0.224 | -1.155 |
| April Mediated Signaling | 1.9 | 0.262 | 0 |
| Aldosterone Signaling in Epithelial Cells | 1.9 | 0.189 | 0 |
| p70S6K Signaling | 1.89 | 0.197 | -0.626 |
| Angiopoietin Signaling | 1.86 | 0.221 | 0 |
| B Cell Activating Factor Signaling | 1.82 | 0.256 | -0.707 |
| Endometrial Cancer Signaling | 1.82 | 0.233 | -1.732 |
| Pyridoxal 5'-phosphate Salvage Pathway | 1.82 | 0.227 | 0 |
| NF-κB Activation by Viruses | 1.81 | 0.218 | -2.668 |
| Spermine Biosynthesis | 1.8 | 1 | #NUM! |
| Glycine Biosynthesis I | 1.8 | 1 | #NUM! |
| Creatine-phosphate Biosynthesis | 1.79 | 0.6 | #NUM! |
| Folate Polyglutamylation | 1.79 | 0.6 | #NUM! |
| Oleate Biosynthesis II (Animals) | 1.77 | 0.385 | 1.342 |
| CCR3 Signaling in Eosinophils | 1.77 | 0.193 | -0.832 |
| Cardiac Hypertrophy Signaling (Enhanced) | 1.75 | 0.157 | -0.956 |
| Mouse Embryonic Stem Cell Pluripotency | 1.74 | 0.202 | -2.683 |
| IL-15 Production | 1.74 | 0.195 | 1.225 |
| AMPK Signaling | 1.74 | 0.174 | 0.6 |
| MYC Mediated Apoptosis Signaling | 1.73 | 0.24 | 0.577 |
| TCA Cycle II (Eukaryotic) | 1.71 | 0.304 | 0.378 |
| Methionine Degradation I (to Homocysteine) | 1.71 | 0.304 | 1.134 |
| Renal Cell Carcinoma Signaling | 1.71 | 0.212 | -1.155 |
| Chemokine Signaling | 1.71 | 0.212 | 0 |
| RHOA Signaling | 1.7 | 0.194 | 0.447 |
| Erythropoietin Signaling Pathway | 1.67 | 0.181 | -0.18 |
| Role of JAK2 in Hormone-like Cytokine Signaling | 1.67 | 0.265 | #NUM! |
| Xenobiotic Metabolism AHR Signaling Pathway | 1.66 | 0.207 | 0.943 |
| Amyloid Processing | 1.66 | 0.235 | -0.378 |
| CD27 Signaling in Lymphocytes | 1.65 | 0.228 | -1.508 |
| Role of CHK Proteins in Cell Cycle Checkpoint Control | 1.65 | 0.228 | 0.302 |
| Neurovascular Coupling Signaling Pathway | 1.64 | 0.173 | -0.48 |
| Fcγ Receptor-mediated Phagocytosis in Macrophages and Monocytes | 1.63 | 0.202 | -0.688 |
| DNA Double-Strand Break Repair by Homologous Recombination | 1.63 | 0.357 | #NUM! |
| ERBB Signaling | 1.63 | 0.202 | -0.943 |
| Telomerase Signaling | 1.61 | 0.196 | -1.414 |
| JAK/STAT Signaling | 1.61 | 0.207 | -0.243 |
| GDNF Family Ligand-Receptor Interactions | 1.6 | 0.211 | -2.496 |
| DNA Methylation and Transcriptional Repression Signaling | 1.59 | 0.257 | #NUM! |
| Role of MAPK Signaling in Inhibiting the Pathogenesis of Influenza | 1.55 | 0.208 | -1.5 |
| nNOS Signaling in Neurons | 1.55 | 0.234 | 0.447 |
| Induction of Apoptosis by HIV1 | 1.54 | 0.215 | -0.832 |
| Role of Macrophages, Fibroblasts and Endothelial Cells in Rheumatoid Arthritis | 1.54 | 0.163 | #NUM! |
| Cysteine Biosynthesis III (mammalia) | 1.52 | 0.28 | 1.134 |
| VEGF Family Ligand-Receptor Interactions | 1.51 | 0.202 | -2.324 |
| Interferon Signaling | 1.51 | 0.25 | -3 |
| Role of Tissue Factor in Cancer | 1.51 | 0.19 | #NUM! |
| CXCR4 Signaling | 1.5 | 0.178 | -0.408 |
| Corticotropin Releasing Hormone Signaling | 1.5 | 0.181 | -1.091 |
| IL-10 Signaling | 1.49 | 0.208 | #NUM! |
| Ephrin B Signaling | 1.49 | 0.208 | 0.832 |
| Netrin Signaling | 1.49 | 0.208 | 1.941 |
| Gap Junction Signaling | 1.45 | 0.172 | #NUM! |
| Pyrimidine Ribonucleotides De Novo Biosynthesis | 1.44 | 0.243 | -1.667 |
| IL-17A Signaling in Gastric Cells | 1.43 | 0.269 | -1.342 |
| Oncostatin M Signaling | 1.43 | 0.233 | -2.333 |
| Antioxidant Action of Vitamin C | 1.42 | 0.188 | 1.886 |
| FLT3 Signaling in Hematopoietic Progenitor Cells | 1.41 | 0.2 | -1.291 |
| Osteoarthritis Pathway | 1.4 | 0.167 | 1.257 |
| Purine Nucleotides De Novo Biosynthesis II | 1.4 | 0.364 | -1 |
| IL-17A Signaling in Fibroblasts | 1.37 | 0.237 | #NUM! |
| Superpathway of Methionine Degradation | 1.37 | 0.237 | 1.667 |
| BER (Base Excision Repair) Pathway | 1.37 | 0.227 | -0.632 |
| FAT10 Cancer Signaling Pathway | 1.36 | 0.22 | -1.265 |
| Guanine and Guanosine Salvage I | 1.36 | 0.667 | #NUM! |
| Methionine Salvage II (Mammalian) | 1.36 | 0.667 | #NUM! |
| S-adenosyl-L-methionine Biosynthesis | 1.36 | 0.667 | #NUM! |
| Synaptic Long Term Depression | 1.34 | 0.169 | 0.707 |
| Superpathway of Serine and Glycine Biosynthesis I | 1.33 | 0.429 | #NUM! |
| Adenine and Adenosine Salvage III | 1.33 | 0.429 | #NUM! |
| Glycoaminoglycan-protein Linkage Region Biosynthesis | 1.33 | 0.429 | #NUM! |
| tRNA Charging | 1.31 | 0.231 | 0.333 |
| Renin-Angiotensin Signaling | 1.3 | 0.18 | -1.091 |

Supplementary Table 2. IPA results for upstream regulators with significant activation z-scores (z-score ≥ 2 or ≤ -2).

| © 2000-2021 QIAGEN. All rights reserved. | | | |  |
| --- | --- | --- | --- | --- |
| **Upstream Regulator** | **Predicted Activation State** | **Activation z-score** | **p-value of overlap** | **Molecule Type** |
| MLXIPL | Inhibited | -6.135 | 1.34E-24 | transcription regulator |
| MYCN | Inhibited | -5.323 | 1.23E-29 | transcription regulator |
| camptothecin | Inhibited | -5.135 | 1.49E-11 | chemical drug |
| Lh | Inhibited | -5.017 | 1.68E-17 | complex |
| prexasertib | Inhibited | -4.646 | 2.99E-07 | chemical drug |
| TCR | Inhibited | -4.471 | 4.15E-07 | complex |
| etoposide | Inhibited | -4.452 | 5.84E-06 | chemical drug |
| Salmonella enterica serotype abortus equi lipopolysaccharide | Inhibited | -4.249 | 4.59E-03 | chemical toxicant |
| poly rI:rC-RNA | Inhibited | -4.116 | 5.71E-05 | biologic drug |
| topotecan | Inhibited | -3.992 | 7.79E-09 | chemical drug |
| NUPR1 | Inhibited | -3.833 | 1.52E-05 | transcription regulator |
| IFNG | Inhibited | -3.675 | 6.57E-05 | cytokine |
| MYC | Inhibited | -3.601 | 3.65E-32 | transcription regulator |
| CREB1 | Inhibited | -3.541 | 2.38E-08 | transcription regulator |
| diclofenac | Inhibited | -3.378 | 2.91E-03 | chemical drug |
| l-asparaginase | Inhibited | -3.327 | 1.22E-05 | biologic drug |
| palbociclib | Inhibited | -3.166 | 5.43E-05 | chemical drug |
| GLI3 | Inhibited | -3.161 | 3.11E-03 | transcription regulator |
| BMP4 | Inhibited | -3.135 | 5.36E-03 | growth factor |
| ATF4 | Inhibited | -3.127 | 1.27E-03 | transcription regulator |
| TAS4464 | Inhibited | -3.118 | 8.46E-03 | chemical drug |
| leukotriene D4 | Inhibited | -3.088 | 1.87E-01 | chemical - endogenous mammalian |
| oblimersen | Inhibited | -3.011 | 1.58E-03 | biologic drug |
| Cdk | Inhibited | -2.983 | 8.46E-03 | group |
| ID3 | Inhibited | -2.969 | 4.85E-02 | transcription regulator |
| mitomycin C | Inhibited | -2.964 | 6.06E-05 | chemical drug |
| Interferon alpha | Inhibited | -2.964 | 4.13E-01 | group |
| butyric acid | Inhibited | -2.934 | 7.22E-06 | chemical - endogenous mammalian |
| FOXO3 | Inhibited | -2.918 | 7.62E-11 | transcription regulator |
| RARB | Inhibited | -2.892 | 4.88E-01 | ligand-dependent nuclear receptor |
| NOD2 | Inhibited | -2.889 | 1.16E-01 | other |
| hydrogen peroxide | Inhibited | -2.87 | 1.43E-06 | chemical - endogenous mammalian |
| IFNA2 | Inhibited | -2.867 | 1.85E-01 | cytokine |
| gentamicin | Inhibited | -2.843 | 1.12E-06 | chemical drug |
| carbamazepine | Inhibited | -2.823 | 1.27E-04 | chemical drug |
| RUNX3 | Inhibited | -2.81 | 2.07E-01 | transcription regulator |
| PDGF BB | Inhibited | -2.768 | 5.78E-10 | complex |
| NCOA3 | Inhibited | -2.744 | 5.55E-03 | transcription regulator |
| CYP19A1 | Inhibited | -2.738 | 1.71E-01 | enzyme |
| IRF1 | Inhibited | -2.737 | 4.93E-01 | transcription regulator |
| PAX5 | Inhibited | -2.728 | 1.02E-02 | transcription regulator |
| PAF1 | Inhibited | -2.714 | 9.77E-03 | other |
| PD173074 | Inhibited | -2.691 | 1.28E-02 | chemical reagent |
| HSP90B1 | Inhibited | -2.684 | 1.02E-02 | other |
| LDB1 | Inhibited | -2.661 | 1.75E-02 | transcription regulator |
| LMO2 | Inhibited | -2.661 | 1.86E-02 | transcription regulator |
| MARK2 | Inhibited | -2.646 | 6.24E-02 | kinase |
| tosedostat | Inhibited | -2.646 | 9.33E-02 | chemical drug |
| asciminib | Inhibited | -2.644 | 5.05E-07 | chemical drug |
| EIF4E | Inhibited | -2.64 | 1.06E-06 | translation regulator |
| aristolochic acid I | Inhibited | -2.629 | 1.16E-03 | chemical toxicant |
| JAK1 | Inhibited | -2.629 | 1.06E-01 | kinase |
| TFEB | Inhibited | -2.628 | 4.34E-03 | transcription regulator |
| delta-aminolevulinic acid | Inhibited | -2.619 | 1.05E-03 | chemical - endogenous mammalian |
| RBL1 | Inhibited | -2.614 | 1.14E-05 | transcription regulator |
| ribavirin | Inhibited | -2.611 | 1.86E-01 | chemical drug |
| Ca2+ | Inhibited | -2.595 | 5.39E-02 | chemical - endogenous mammalian |
| SPDEF | Inhibited | -2.585 | 4.61E-04 | transcription regulator |
| temozolomide | Inhibited | -2.563 | 5.53E-03 | chemical drug |
| ASPSCR1-TFE3 | Inhibited | -2.561 | 2.17E-03 | fusion gene/product |
| seocalcitol | Inhibited | -2.56 | 6.96E-03 | chemical drug |
| LCK | Inhibited | -2.538 | 1.64E-01 | kinase |
| FCGR2A | Inhibited | -2.527 | 1.87E-01 | transmembrane receptor |
| RELA | Inhibited | -2.524 | 1.55E-02 | transcription regulator |
| TNFSF10 | Inhibited | -2.506 | 1.69E-02 | cytokine |
| IRF5 | Inhibited | -2.497 | 6.95E-02 | transcription regulator |
| EIF2AK2 | Inhibited | -2.475 | 6.07E-02 | kinase |
| lipoarabinomannan | Inhibited | -2.449 | 2.55E-02 | chemical - endogenous non-mammalian |
| HOXA5 | Inhibited | -2.449 | 8.85E-02 | transcription regulator |
| Z36 | Inhibited | -2.449 | 1.39E-01 | chemical reagent |
| I-BET-151 | Inhibited | -2.449 | 4.16E-01 | chemical reagent |
| NFkB (complex) | Inhibited | -2.446 | 1.10E-02 | complex |
| propylthiouracil | Inhibited | -2.443 | 1.07E-02 | chemical drug |
| CLOCK | Inhibited | -2.441 | 3.46E-14 | transcription regulator |
| BMP | Inhibited | -2.433 | 5.77E-02 | group |
| cis-urocanic acid | Inhibited | -2.425 | 1.99E-02 | chemical drug |
| SLC27A2 | Inhibited | -2.425 | 5.14E-01 | transporter |
| RAF1 | Inhibited | -2.422 | 4.66E-04 | kinase |
| methyl methanesulfonate | Inhibited | -2.421 | 1.18E-03 | chemical toxicant |
| doxorubicin | Inhibited | -2.402 | 1.20E-08 | chemical drug |
| Fc gamma receptor | Inhibited | -2.4 | 1.23E-01 | group |
| NFATC4 | Inhibited | -2.397 | 1.31E-02 | transcription regulator |
| TICAM1 | Inhibited | -2.397 | 1.07E-01 | other |
| carrageenan | Inhibited | -2.391 | 7.63E-02 | chemical drug |
| TLR7 | Inhibited | -2.374 | 5.24E-01 | transmembrane receptor |
| NOSTRIN | Inhibited | -2.369 | 6.68E-03 | transcription regulator |
| IFI16 | Inhibited | -2.356 | 1.40E-03 | transcription regulator |
| ELF4 | Inhibited | -2.355 | 1.94E-02 | transcription regulator |
| methylnitronitrosoguanidine | Inhibited | -2.352 | 3.01E-03 | chemical toxicant |
| hydroxyurea | Inhibited | -2.351 | 2.69E-03 | chemical drug |
| TRADD | Inhibited | -2.345 | 8.09E-03 | other |
| carbamylcholine | Inhibited | -2.34 | 1.02E-04 | chemical drug |
| CX-5461 | Inhibited | -2.333 | 1.78E-03 | chemical drug |
| KAT6A | Inhibited | -2.328 | 2.43E-03 | enzyme |
| MAPK7 | Inhibited | -2.319 | 1.99E-05 | kinase |
| NDRG1 | Inhibited | -2.317 | 2.04E-02 | kinase |
| P38 MAPK | Inhibited | -2.316 | 7.34E-03 | group |
| ECSIT | Inhibited | -2.287 | 1.25E-05 | transcription regulator |
| ABT-737 | Inhibited | -2.286 | 5.03E-02 | chemical drug |
| IKBKB | Inhibited | -2.269 | 4.83E-02 | kinase |
| thapsigargin | Inhibited | -2.265 | 2.03E-05 | chemical toxicant |
| GDF2 | Inhibited | -2.25 | 1.11E-04 | growth factor |
| amitriptyline | Inhibited | -2.236 | 2.31E-01 | chemical drug |
| UBQLN2 | Inhibited | -2.219 | 6.51E-03 | other |
| HFE | Inhibited | -2.213 | 1.81E-01 | transmembrane receptor |
| mir-103 | Inhibited | -2.208 | 1.21E-01 | microRNA |
| CGAS | Inhibited | -2.208 | 2.68E-01 | enzyme |
| Firre | Inhibited | -2.2 | 3.80E-05 | other |
| olaparib | Inhibited | -2.2 | 9.45E-03 | chemical drug |
| TERF2IP | Inhibited | -2.197 | 1.58E-01 | other |
| IL12 (family) | Inhibited | -2.195 | 2.49E-01 | group |
| CP-55940 | Inhibited | -2.191 | 2.33E-02 | chemical reagent |
| LYL1 | Inhibited | -2.186 | 2.43E-02 | transcription regulator |
| dalfampridine | Inhibited | -2.178 | 4.21E-01 | chemical drug |
| norepinephrine | Inhibited | -2.177 | 3.19E-01 | chemical - endogenous mammalian |
| STING1 | Inhibited | -2.169 | 4.34E-01 | other |
| mir-218 | Inhibited | -2.168 | 1.16E-01 | microRNA |
| zoledronic acid | Inhibited | -2.157 | 8.56E-02 | chemical drug |
| FGF9 | Inhibited | -2.156 | 2.84E-01 | growth factor |
| FOXO4 | Inhibited | -2.155 | 1.00E-05 | transcription regulator |
| bicuculline | Inhibited | -2.155 | 2.18E-01 | chemical - endogenous non-mammalian |
| RND3 | Inhibited | -2.153 | 8.19E-04 | enzyme |
| 3,3'-diindolylmethane | Inhibited | -2.146 | 1.04E-03 | chemical drug |
| IFNL1 | Inhibited | -2.144 | 4.73E-01 | cytokine |
| sodium chloride | Inhibited | -2.142 | 2.46E-01 | chemical - endogenous mammalian |
| GF 120918 | Inhibited | -2.138 | 3.87E-03 | chemical reagent |
| Pkc(s) | Inhibited | -2.13 | 2.07E-03 | group |
| DDIT3 | Inhibited | -2.126 | 5.50E-04 | transcription regulator |
| actinonin | Inhibited | -2.121 | 7.56E-04 | chemical reagent |
| ADORA2B | Inhibited | -2.121 | 1.08E-01 | G-protein coupled receptor |
| mir-1 | Inhibited | -2.097 | 5.76E-05 | microRNA |
| IFNGR1 | Inhibited | -2.091 | 1.87E-01 | transmembrane receptor |
| Ifn | Inhibited | -2.086 | 3.52E-01 | group |
| SP2509 | Inhibited | -2.083 | 2.17E-13 | chemical reagent |
| HAVCR1 | Inhibited | -2.083 | 3.46E-03 | other |
| KDM3B | Inhibited | -2.065 | 1.33E-05 | enzyme |
| IL33 | Inhibited | -2.061 | 9.12E-02 | cytokine |
| mir-122 | Inhibited | -2.056 | 1.35E-04 | microRNA |
| corticosterone | Inhibited | -2.024 | 7.56E-02 | chemical - endogenous mammalian |
| stallimycin | Inhibited | -2.017 | 7.60E-02 | biologic drug |
| bromodeoxyuridine | Inhibited | -2.017 | 1.58E-01 | chemical drug |
| Il3 | Inhibited | -2.016 | 1.25E-01 | cytokine |
| KMT2A | Inhibited | -2.013 | 1.12E-01 | transcription regulator |
| lenalidomide | Inhibited | -2.01 | 8.49E-05 | chemical drug |
| NEDD9 | Inhibited | -2.01 | 4.00E-01 | other |
| PML | Inhibited | -2.008 | 1.83E-05 | transcription regulator |
| GnRH-A | Inhibited | -2.001 | 8.39E-04 | chemical reagent |
| ST3-Hel2A-2 | Inhibited | -2 | 4.72E-02 | chemical reagent |
| orlistat | Inhibited | -2 | 4.72E-02 | chemical drug |
| idarubicin | Inhibited | -2 | 5.03E-02 | chemical drug |
| PLCE1 | Inhibited | -2 | 1.04E-01 | enzyme |
| tetrodotoxin | Inhibited | -2 | 1.71E-01 | chemical drug |
| CHD1 | Inhibited | -2 | 1.80E-01 | enzyme |
| SMYD3 | Inhibited | -2 | 2.09E-01 | enzyme |
| NFAT (complex) | Inhibited | -2 | 4.90E-01 | complex |
| ST1926 | Activated | 2 | 1.39E-10 | chemical drug |
| FLVCR1 | Activated | 2 | 1.38E-03 | transporter |
| SOX7 | Activated | 2 | 1.28E-02 | transcription regulator |
| NELFB | Activated | 2 | 6.35E-02 | other |
| Pdgfr | Activated | 2 | 8.24E-02 | group |
| MBTPS1 | Activated | 2 | 8.24E-02 | peptidase |
| NBEAL2 | Activated | 2 | 1.80E-01 | other |
| alpha-amanitin | Activated | 2 | 2.09E-01 | chemical toxicant |
| pexidartinib | Activated | 2 | 2.35E-01 | chemical drug |
| AURKB | Activated | 2 | 2.70E-01 | kinase |
| DUSP5 | Activated | 2 | 2.70E-01 | phosphatase |
| Nppb | Activated | 2 | 4.28E-01 | other |
| GW7647 | Activated | 2 | 4.90E-01 | chemical drug |
| HOXB13 | Activated | 2 | 5.20E-01 | transcription regulator |
| bezafibrate | Activated | 2.005 | 4.11E-01 | chemical drug |
| MTDH | Activated | 2.016 | 4.88E-02 | transcription regulator |
| POR | Activated | 2.08 | 2.18E-01 | enzyme |
| THRB | Activated | 2.088 | 1.47E-01 | ligand-dependent nuclear receptor |
| CAV1 | Activated | 2.096 | 3.34E-03 | transmembrane receptor |
| bisindolylmaleimide I | Activated | 2.102 | 4.80E-05 | chemical drug |
| TREM2 | Activated | 2.102 | 3.85E-01 | transmembrane receptor |
| DEF6 | Activated | 2.105 | 7.32E-03 | other |
| pyridaben | Activated | 2.111 | 1.05E-01 | chemical toxicant |
| maneb | Activated | 2.111 | 2.08E-01 | chemical toxicant |
| mir-154 | Activated | 2.112 | 4.80E-02 | microRNA |
| FOXM1 | Activated | 2.118 | 1.29E-05 | transcription regulator |
| KLF2 | Activated | 2.118 | 7.30E-04 | transcription regulator |
| NFU1 | Activated | 2.121 | 1.37E-01 | other |
| IRGM | Activated | 2.135 | 3.64E-01 | enzyme |
| Abcb1b | Activated | 2.137 | 5.39E-03 | transporter |
| ABCB1 | Activated | 2.137 | 1.58E-01 | transporter |
| TWNK | Activated | 2.138 | 7.32E-03 | enzyme |
| chelerythrine | Activated | 2.138 | 9.72E-03 | chemical drug |
| SU6656 | Activated | 2.138 | 3.53E-02 | chemical toxicant |
| H2AZ1 | Activated | 2.138 | 4.20E-02 | other |
| LG100268 | Activated | 2.144 | 4.41E-01 | chemical reagent |
| QKI | Activated | 2.157 | 2.91E-04 | other |
| IFRD1 | Activated | 2.157 | 4.50E-02 | other |
| MAP3K12 | Activated | 2.168 | 5.54E-06 | kinase |
| sulfasalazine | Activated | 2.169 | 1.16E-01 | chemical drug |
| allopurinol | Activated | 2.177 | 6.07E-02 | chemical drug |
| ASAH1 | Activated | 2.178 | 5.67E-02 | enzyme |
| pyrrolidine dithiocarbamate | Activated | 2.186 | 1.01E-01 | chemical reagent |
| LRP5 | Activated | 2.19 | 3.91E-01 | transmembrane receptor |
| COMMD3-BMI1 | Activated | 2.191 | 2.58E-02 | transcription regulator |
| CASP3 | Activated | 2.2 | 2.00E-01 | peptidase |
| NFE2L2 | Activated | 2.211 | 1.10E-02 | transcription regulator |
| ebselen | Activated | 2.213 | 2.91E-02 | chemical drug |
| HSF2 | Activated | 2.213 | 1.22E-01 | transcription regulator |
| ARRB2 | Activated | 2.218 | 4.16E-01 | other |
| nimodipine | Activated | 2.219 | 6.43E-02 | chemical drug |
| SMURF2 | Activated | 2.219 | 9.68E-02 | enzyme |
| pifithrin alpha | Activated | 2.219 | 1.81E-01 | chemical reagent |
| POLR2M | Activated | 2.219 | 2.31E-01 | other |
| PLD1 | Activated | 2.222 | 2.05E-01 | enzyme |
| RV 538 | Activated | 2.224 | 2.09E-02 | chemical reagent |
| SKP2 | Activated | 2.225 | 9.39E-04 | other |
| PCLAF | Activated | 2.234 | 2.09E-02 | other |
| MNT | Activated | 2.236 | 3.30E-04 | transcription regulator |
| ALKBH1 | Activated | 2.236 | 1.62E-03 | enzyme |
| NSUN3 | Activated | 2.236 | 1.62E-03 | enzyme |
| Pde | Activated | 2.236 | 3.24E-03 | group |
| CRY1 | Activated | 2.236 | 2.09E-02 | enzyme |
| RAE1 | Activated | 2.236 | 9.68E-02 | other |
| lapatinib/pazopanib | Activated | 2.236 | 1.16E-01 | chemical drug |
| KN-62 | Activated | 2.236 | 2.57E-01 | chemical drug |
| CCR1 | Activated | 2.236 | 3.11E-01 | G-protein coupled receptor |
| CITED2 | Activated | 2.242 | 2.94E-01 | transcription regulator |
| estradiol benzoate | Activated | 2.247 | 5.16E-01 | chemical drug |
| miR-92a-3p (and other miRNAs w/seed AUUGCAC) | Activated | 2.258 | 1.66E-03 | mature microRNA |
| FOXC2 | Activated | 2.259 | 1.19E-02 | transcription regulator |
| UCHL1 | Activated | 2.261 | 2.24E-02 | peptidase |
| Focal adhesion kinase | Activated | 2.28 | 4.50E-02 | group |
| E2f | Activated | 2.291 | 6.42E-05 | group |
| IgG | Activated | 2.293 | 9.32E-04 | complex |
| PAK2 | Activated | 2.309 | 2.79E-03 | kinase |
| glutathione | Activated | 2.338 | 1.92E-02 | chemical - endogenous mammalian |
| SMARCA2 | Activated | 2.356 | 8.83E-03 | transcription regulator |
| CKAP2L | Activated | 2.357 | 9.68E-05 | other |
| 2-methoxyestradiol | Activated | 2.36 | 2.41E-01 | chemical - endogenous mammalian |
| propranolol | Activated | 2.39 | 2.98E-01 | chemical drug |
| 2-aminopurine | Activated | 2.392 | 8.85E-02 | chemical reagent |
| RABL6 | Activated | 2.393 | 1.88E-03 | other |
| IL1RN | Activated | 2.395 | 3.75E-01 | cytokine |
| valsartan | Activated | 2.4 | 2.86E-01 | chemical drug |
| cyanocobalamin | Activated | 2.401 | 1.39E-01 | chemical - endogenous mammalian |
| IL10 | Activated | 2.408 | 2.26E-01 | cytokine |
| methylselenic acid | Activated | 2.425 | 1.89E-13 | chemical reagent |
| PACS1 | Activated | 2.425 | 9.30E-03 | other |
| SENP7 | Activated | 2.433 | 1.19E-04 | peptidase |
| GABA | Activated | 2.435 | 2.02E-18 | chemical - endogenous mammalian |
| BDNF | Activated | 2.448 | 5.13E-03 | growth factor |
| SIN3A | Activated | 2.449 | 1.39E-02 | transcription regulator |
| IKZF2 | Activated | 2.449 | 3.66E-01 | transcription regulator |
| KCNK9 | Activated | 2.449 | 4.66E-01 | ion channel |
| MITF | Activated | 2.455 | 7.76E-05 | transcription regulator |
| ESR2 | Activated | 2.457 | 4.64E-12 | ligand-dependent nuclear receptor |
| NLRX1 | Activated | 2.534 | 1.19E-01 | other |
| GPS2 | Activated | 2.537 | 4.15E-01 | transcription regulator |
| miR-27a-3p (and other miRNAs w/seed UCACAGU) | Activated | 2.538 | 1.31E-01 | mature microRNA |
| MAX | Activated | 2.547 | 2.08E-02 | transcription regulator |
| TBX2 | Activated | 2.558 | 2.50E-04 | transcription regulator |
| DSCAM | Activated | 2.558 | 2.70E-03 | other |
| AGT | Activated | 2.586 | 6.03E-06 | growth factor |
| mir-25 | Activated | 2.588 | 2.98E-01 | microRNA |
| SOD2 | Activated | 2.621 | 4.06E-01 | enzyme |
| TGFBR2 | Activated | 2.656 | 2.64E-04 | kinase |
| MKNK1 | Activated | 2.667 | 1.86E-03 | kinase |
| PD98059 | Activated | 2.67 | 7.12E-06 | chemical - kinase inhibitor |
| GNA12 | Activated | 2.735 | 5.35E-02 | enzyme |
| ERG | Activated | 2.779 | 4.20E-08 | transcription regulator |
| MYBL2 | Activated | 2.862 | 3.88E-04 | transcription regulator |
| ETV6-RUNX1 | Activated | 2.975 | 4.23E-03 | fusion gene/product |
| sirolimus | Activated | 3.053 | 1.00E-12 | chemical drug |
| TAL1 | Activated | 3.278 | 1.71E-03 | transcription regulator |
| GATA4 | Activated | 3.281 | 5.23E-02 | transcription regulator |
| RICTOR | Activated | 3.299 | 7.18E-10 | other |
| CD24 | Activated | 3.823 | 7.34E-07 | other |
| HSF1 | Activated | 4.006 | 9.31E-07 | transcription regulator |
| ESR1 | Activated | 4.471 | 3.57E-18 | ligand-dependent nuclear receptor |
| COPS5 | Activated | 4.609 | 9.66E-13 | transcription regulator |
| LARP1 | Activated | 7.433 | 5.59E-38 | translation regulator |
