# Supplementary material for: Targeting STAU1 prevents p53 apoptotic signaling in neurodegeneration
Source: Cell Death Dis. 2025 Oct 27;16(1):761. doi: 10.1038/s41419-025-08067-0 (PMC12559333; doi:10.1038/s41419-025-08067-0)

Fig. 3A

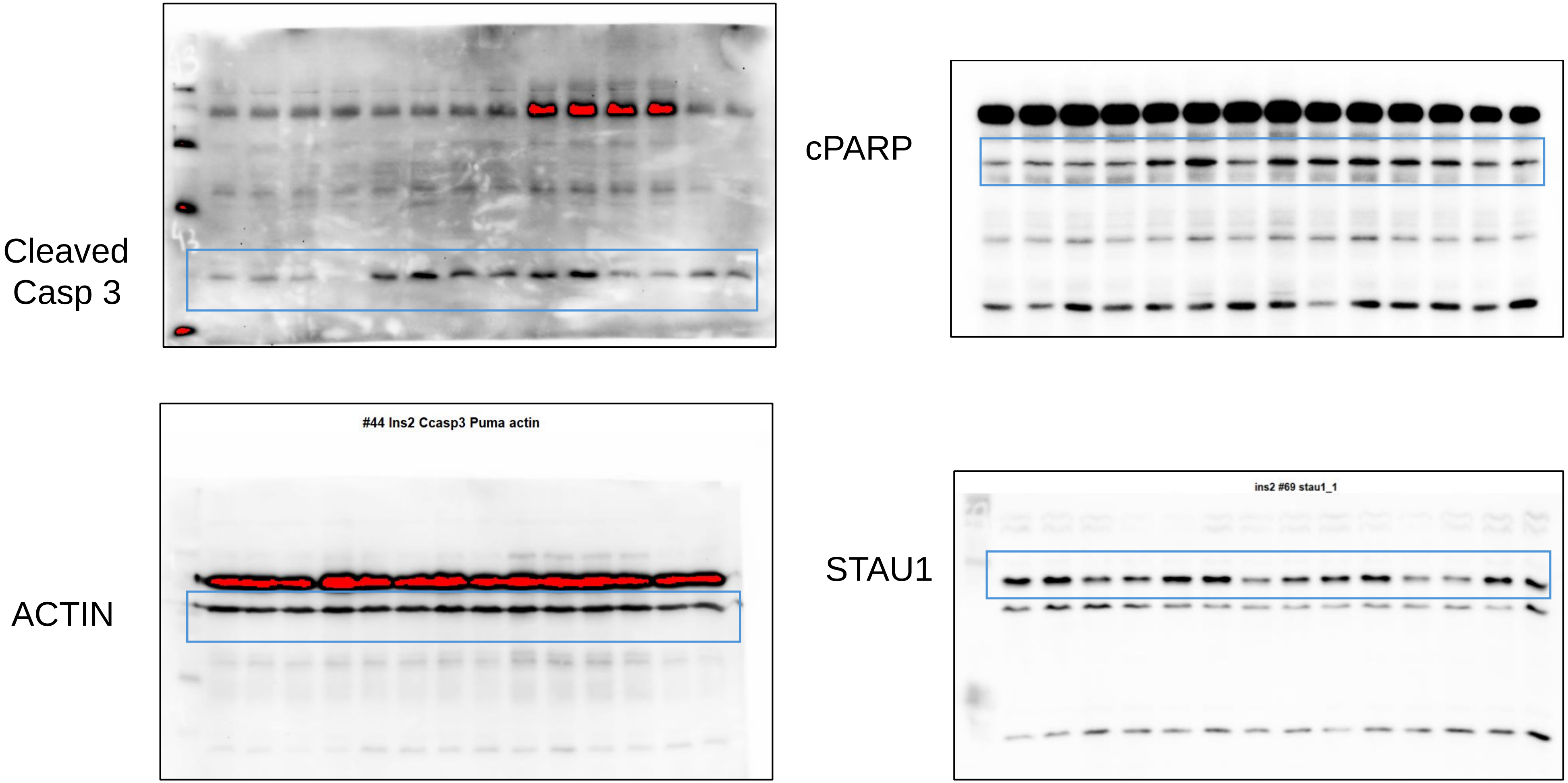
Cleaved Casp 3
cPARP
#44 Ins2 Ccasp3 Puma actin
ACTIN
ins2 #69 stau1_1
STAU1

Fig. 3B

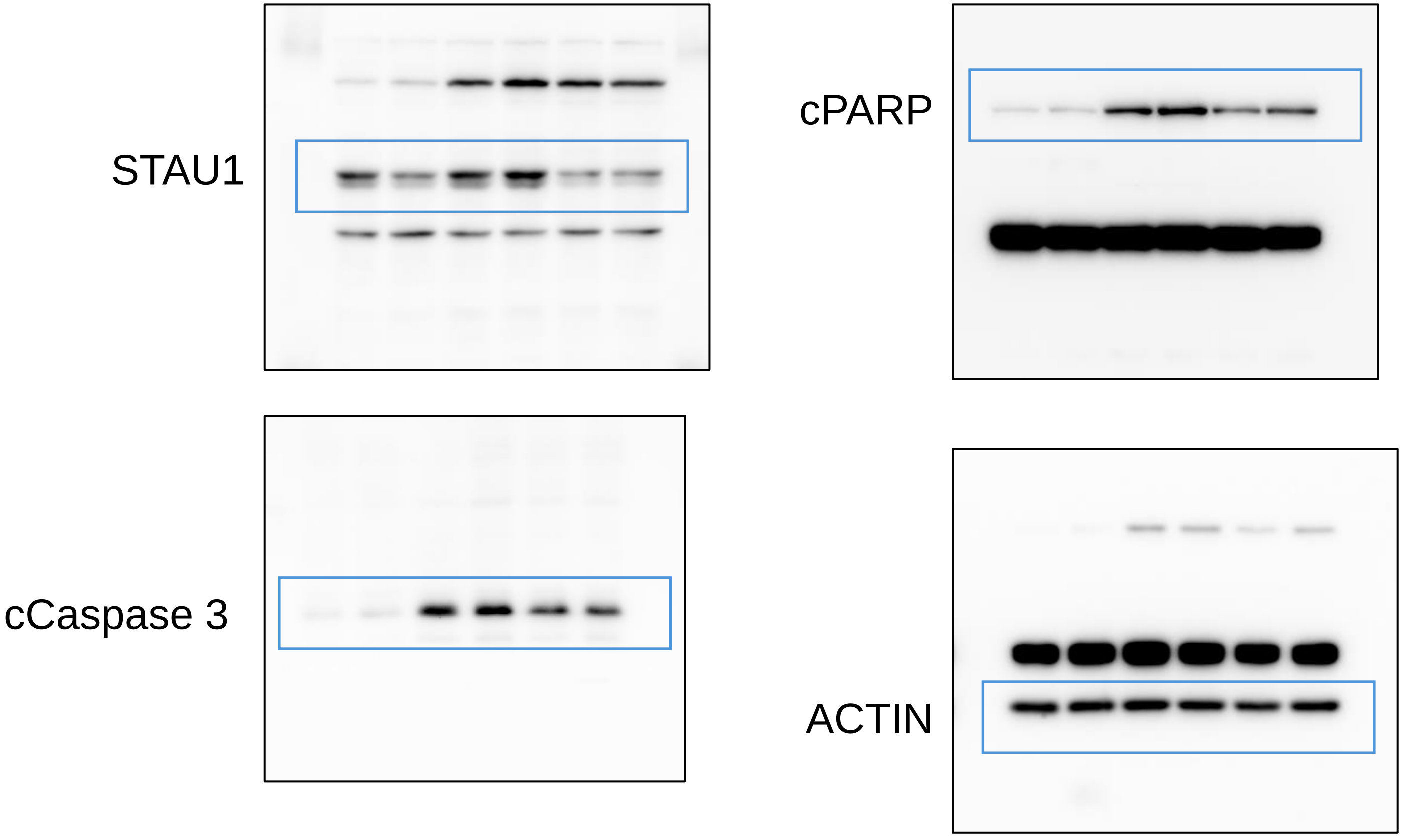

Fig. 3C

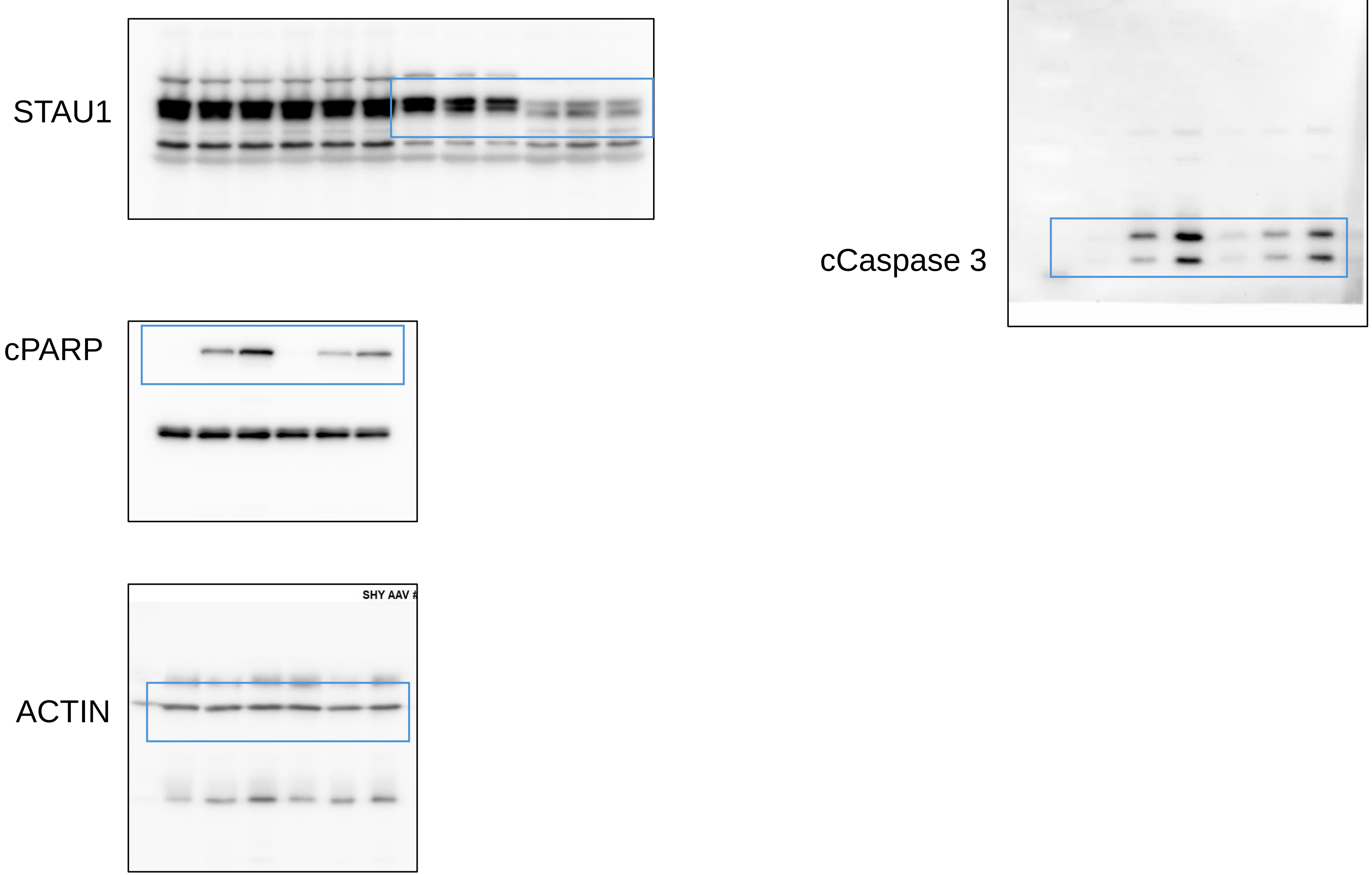

Fig. 3C

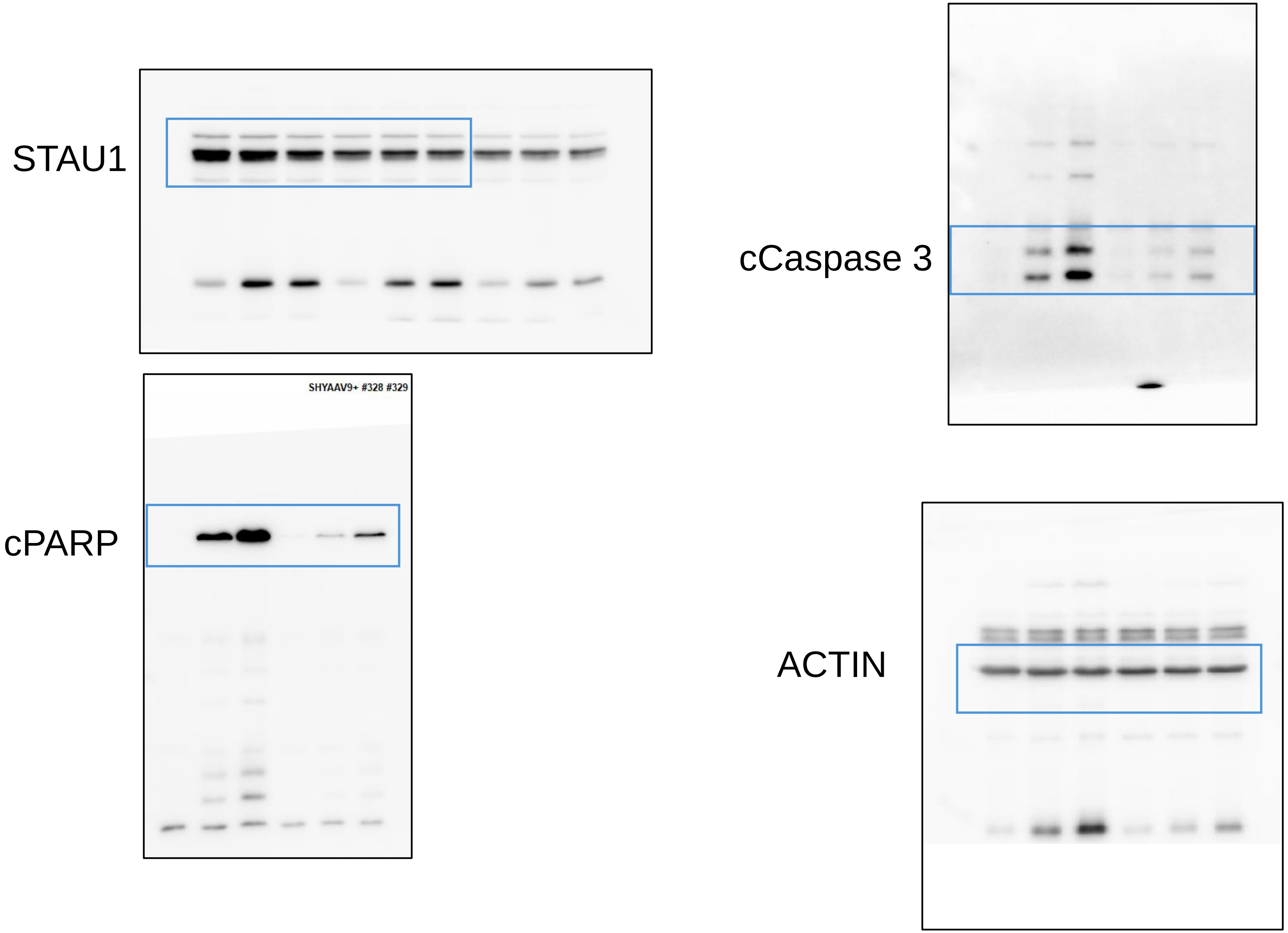

Fig. 3D

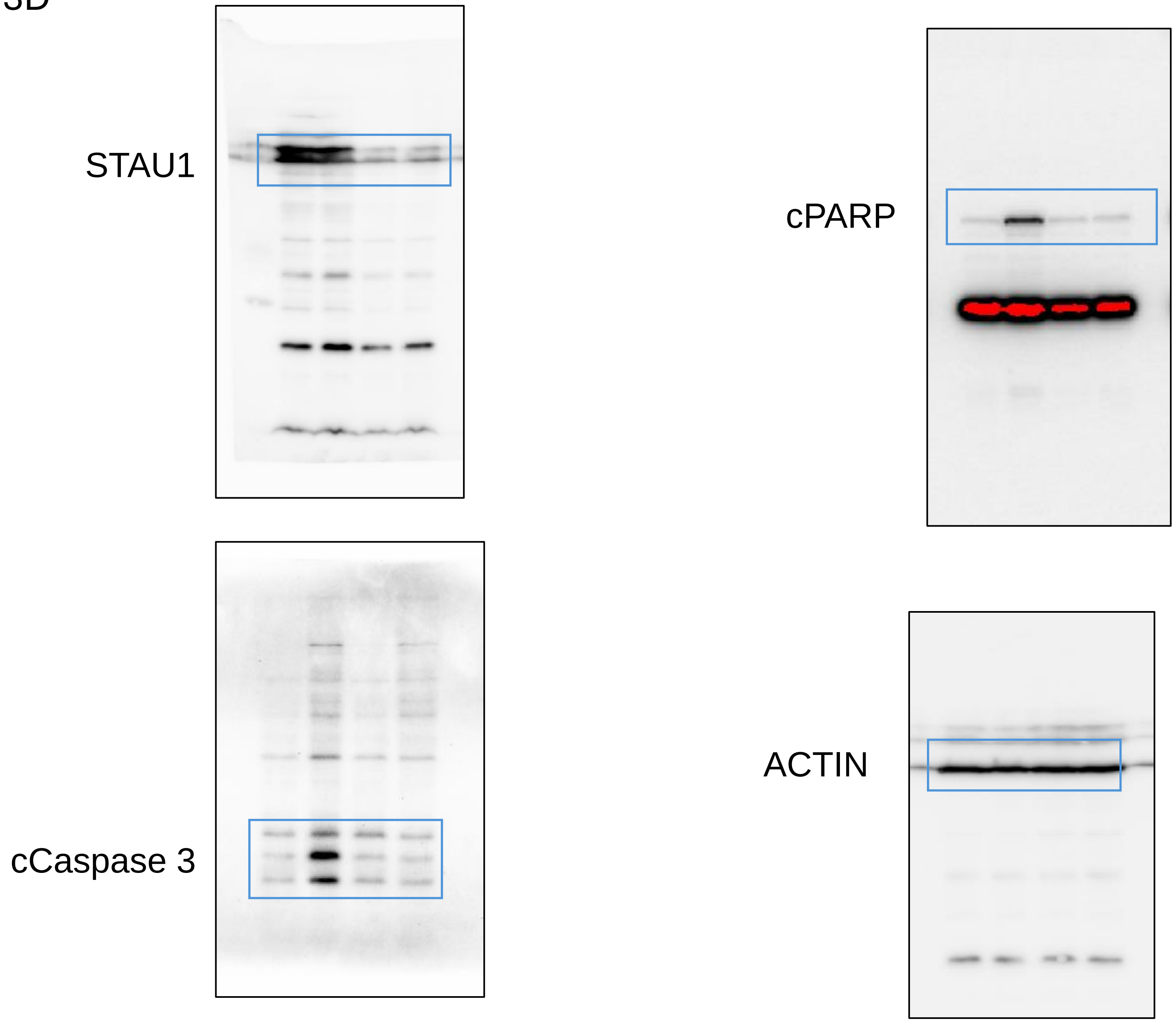

Fig. 3E

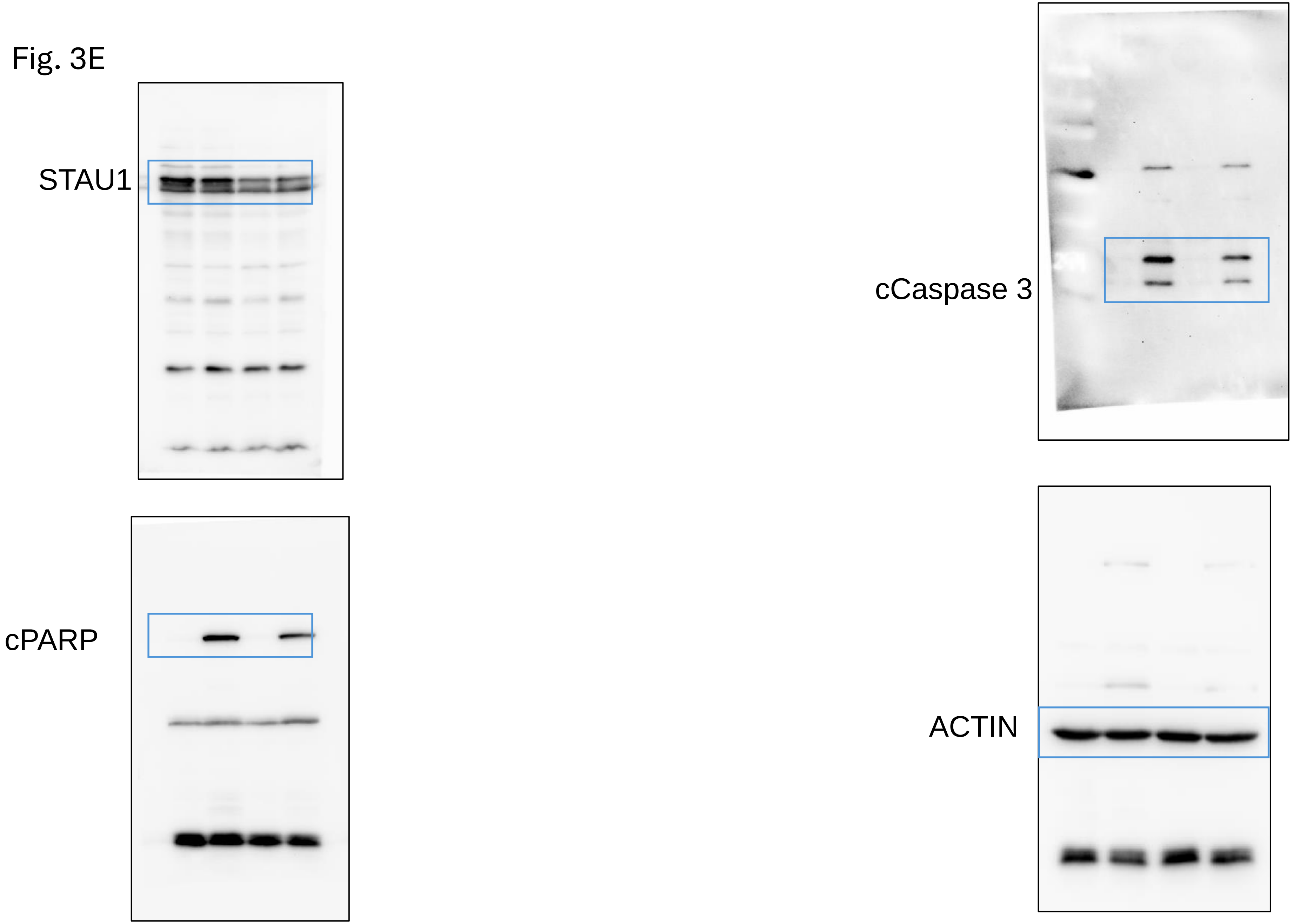

Fig. 3F

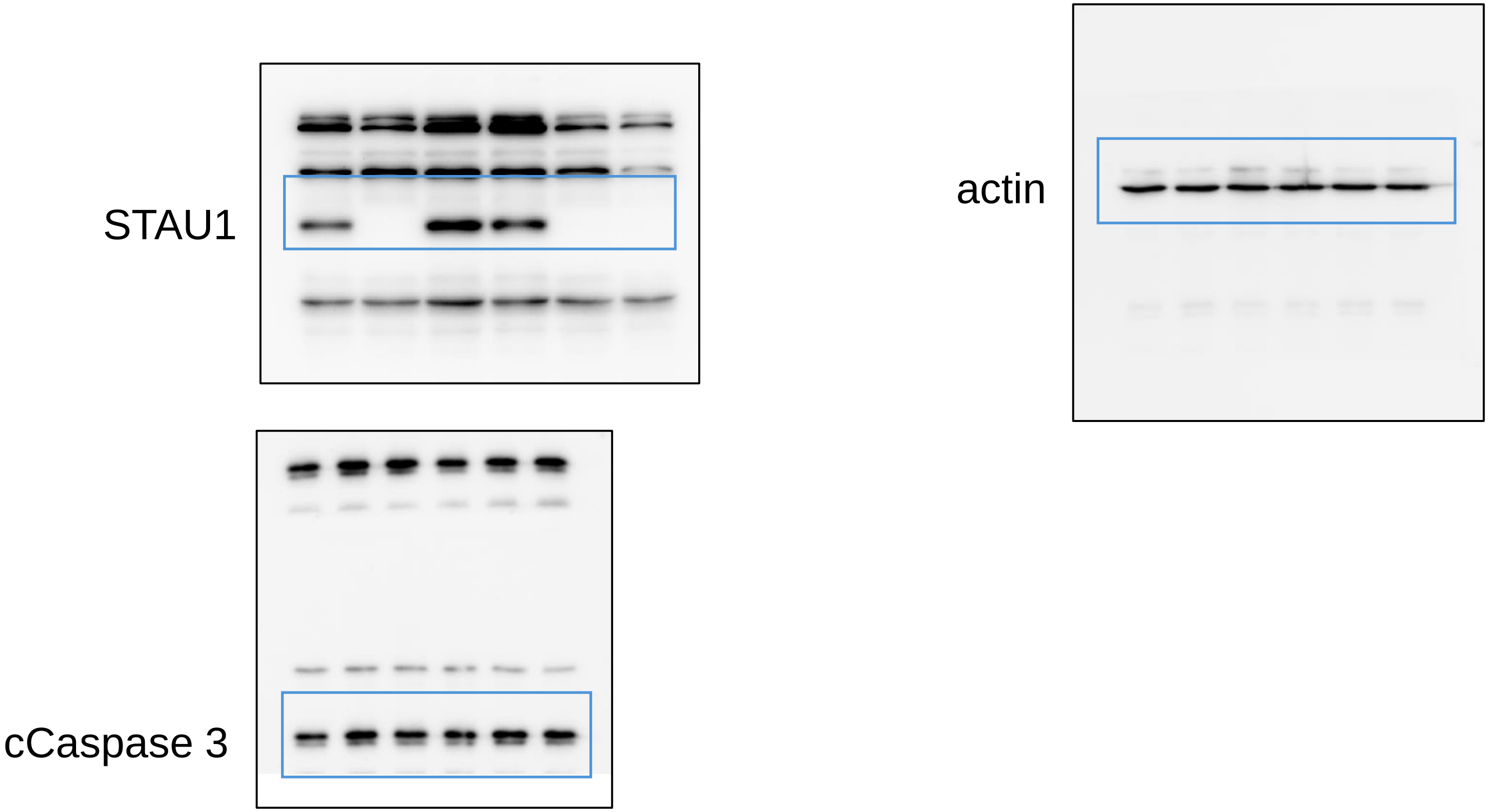

Fig. 3A

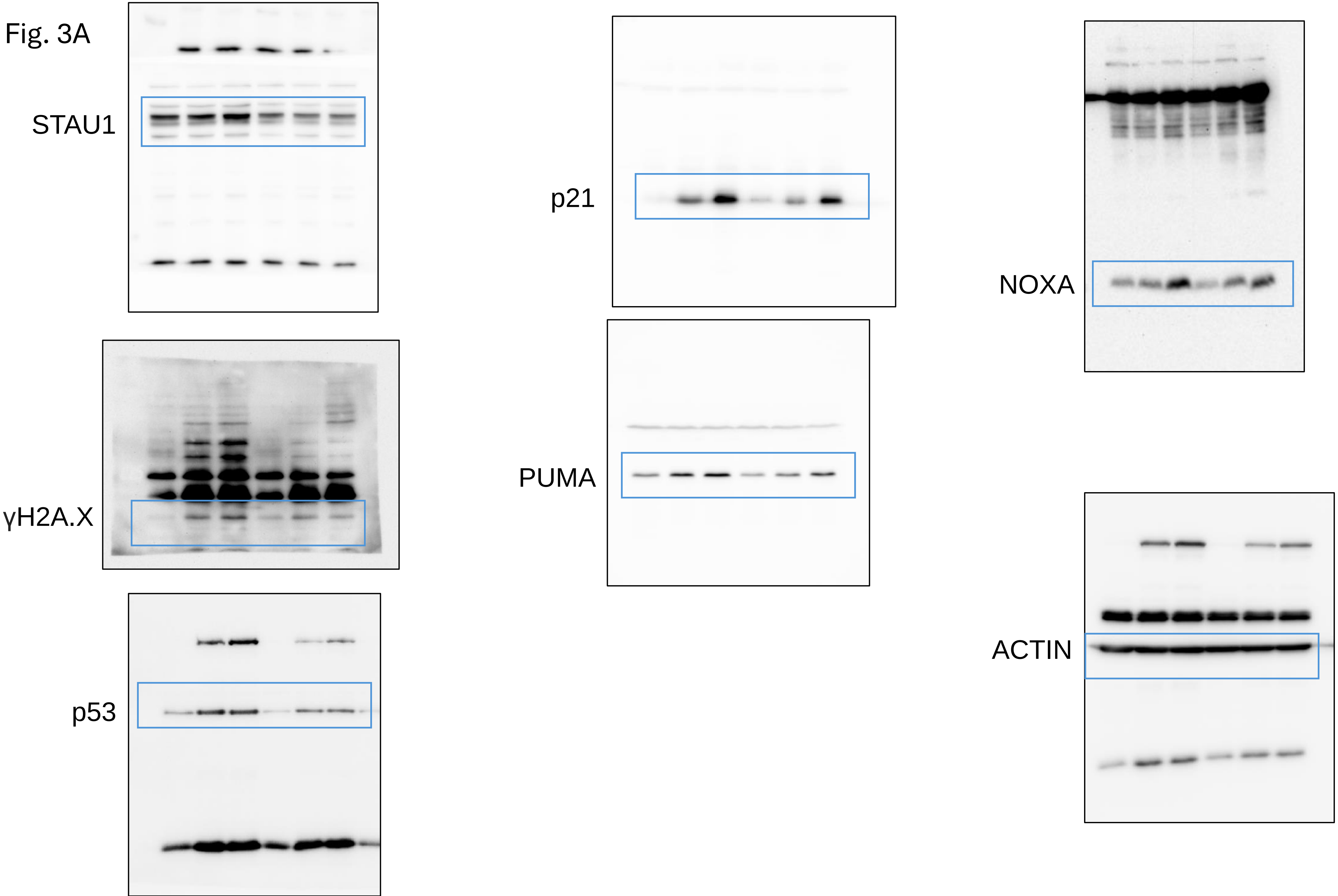

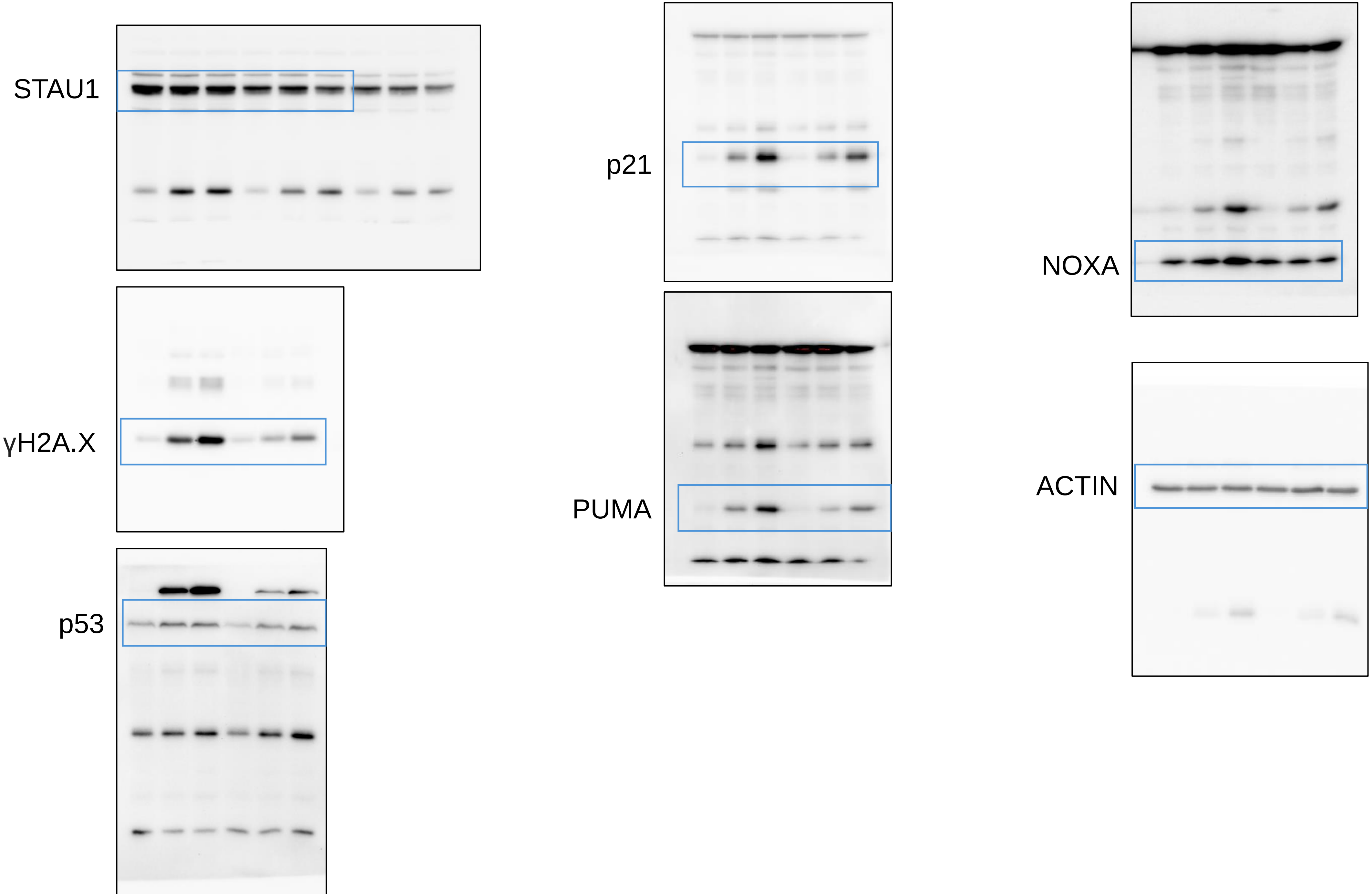
STAU1
γH2A.X
p53
p21
PUMA
NOXA
ACTIN

Fig. 4B

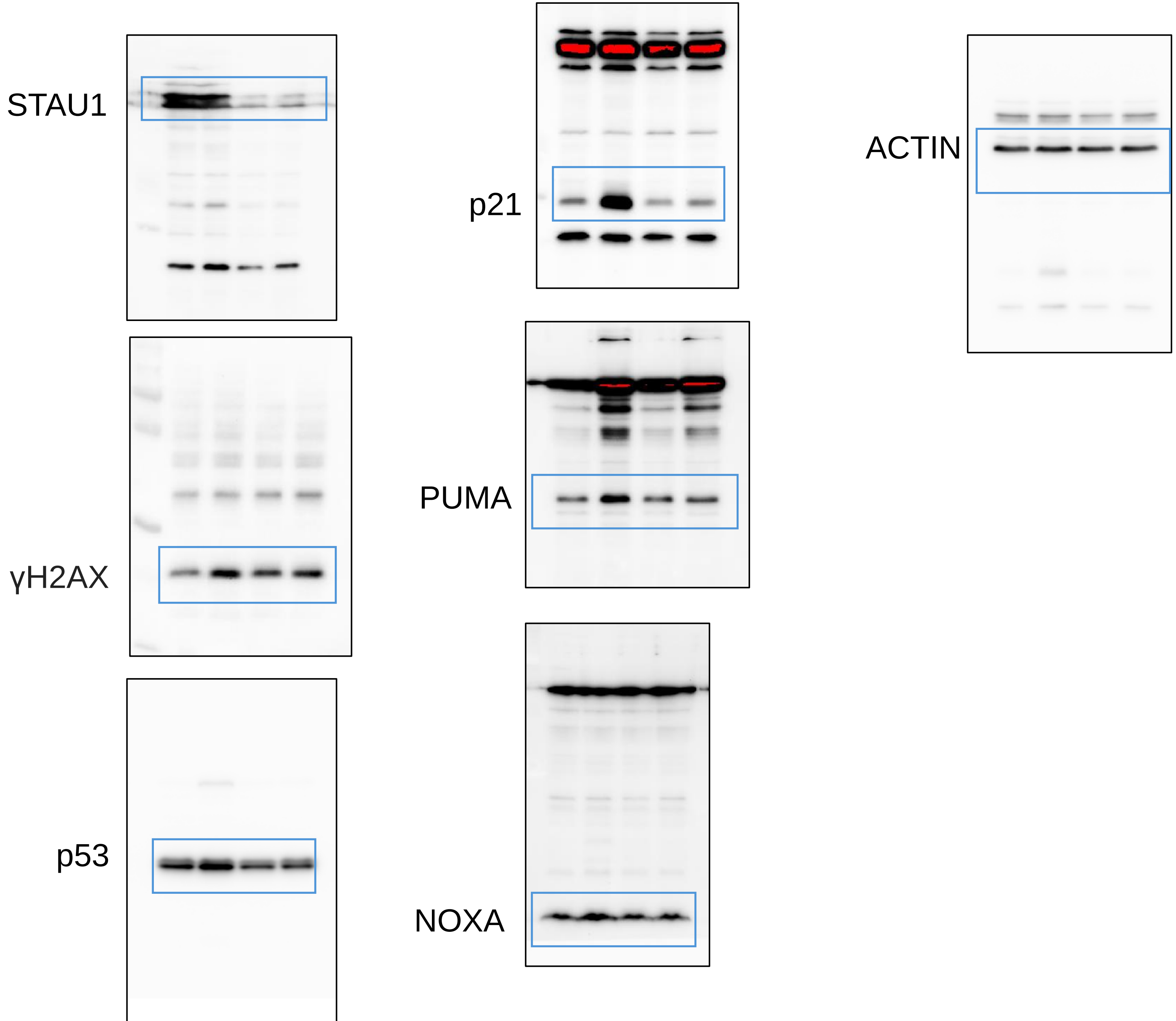

Fig. 4B

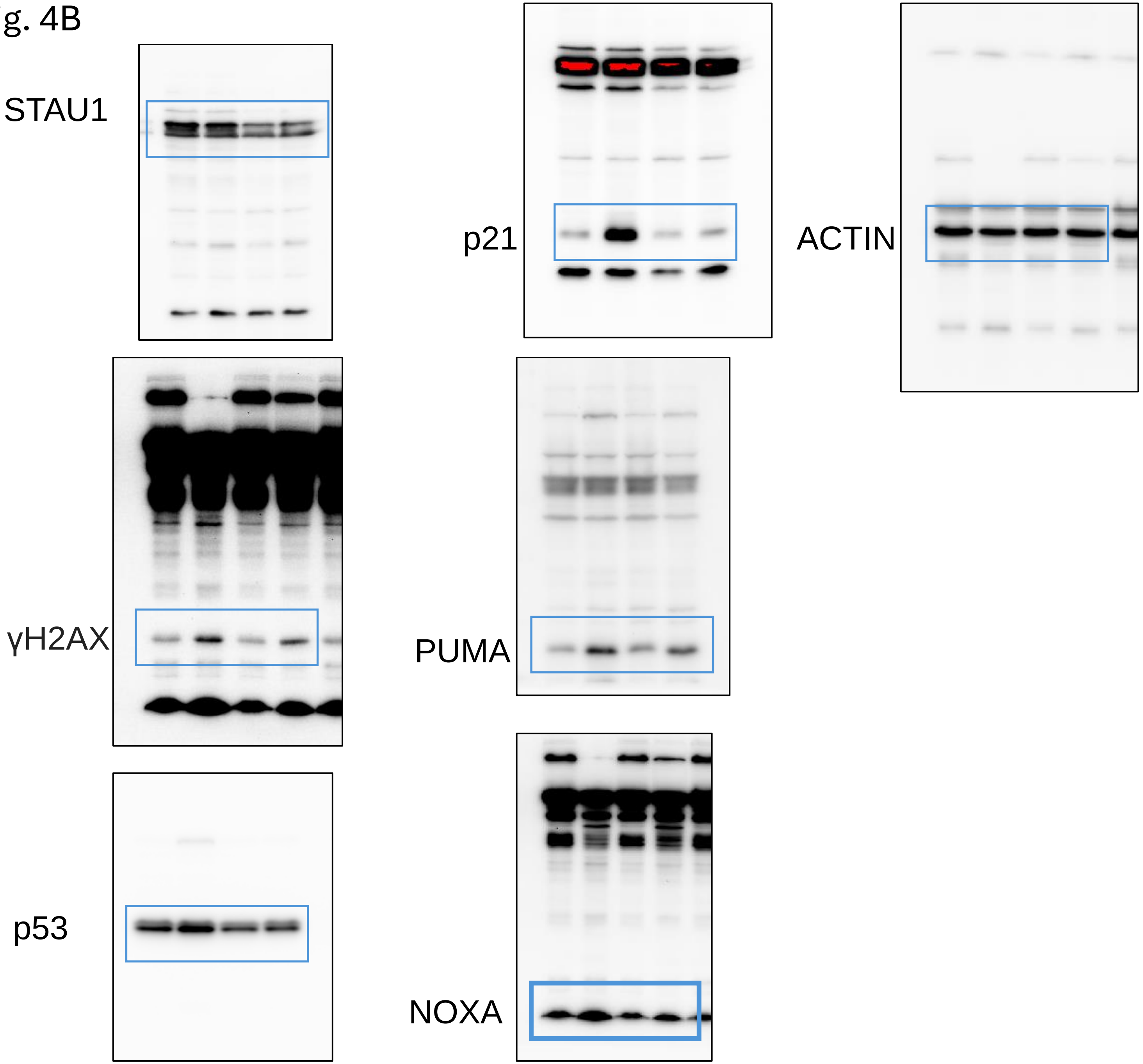

Fig. 4C

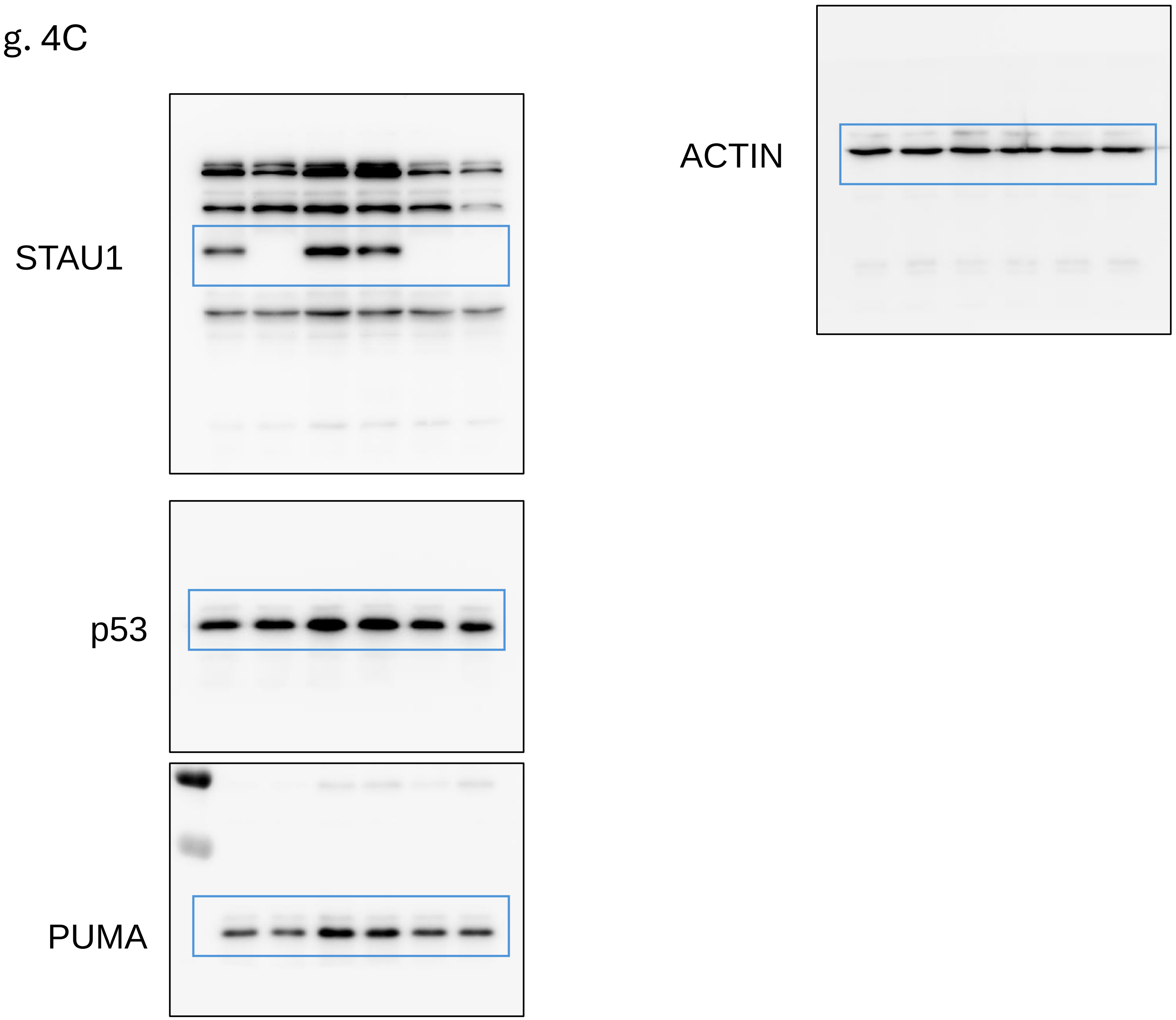

Fig. 5A

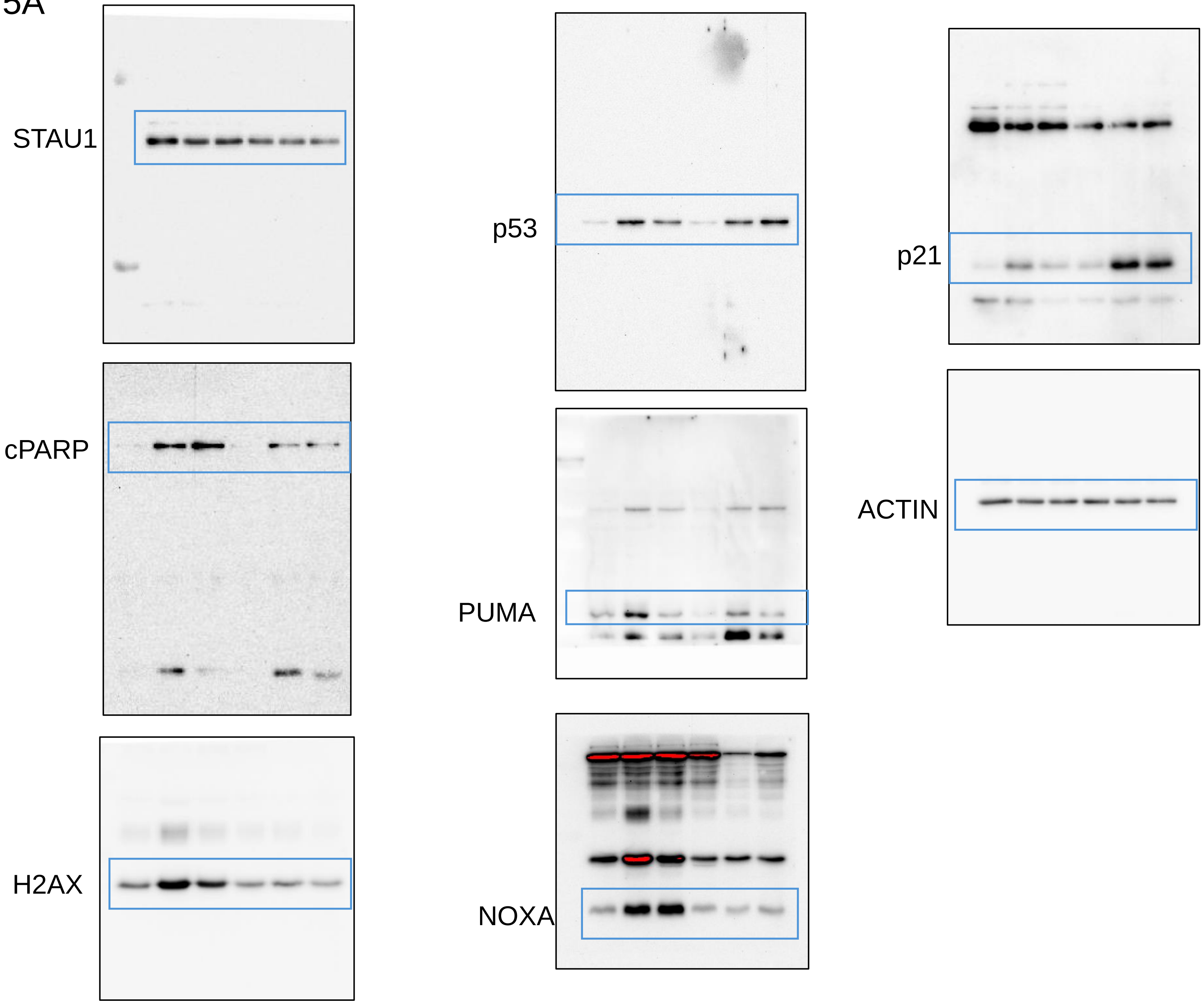

Fig. 5D

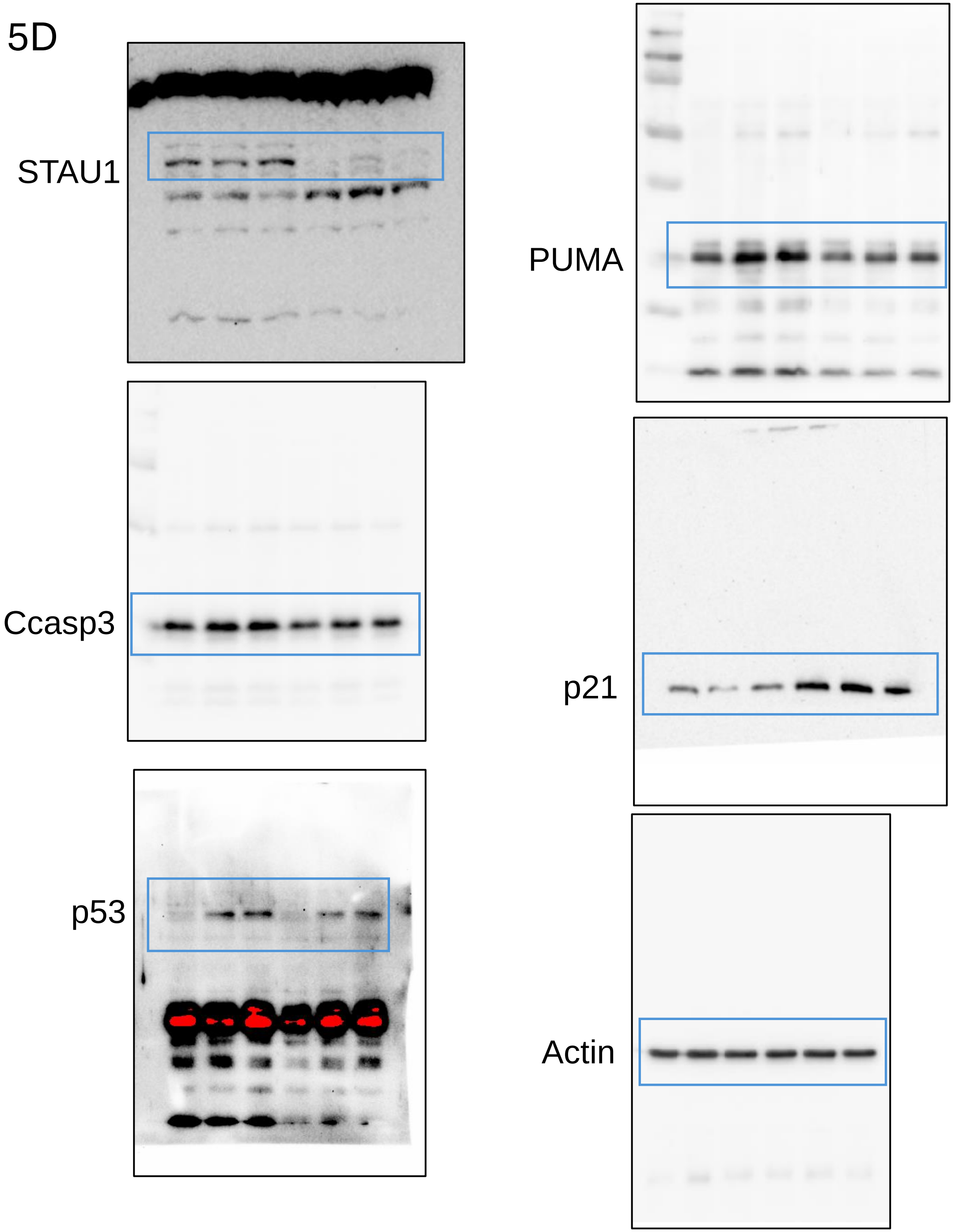

Fig. 6A

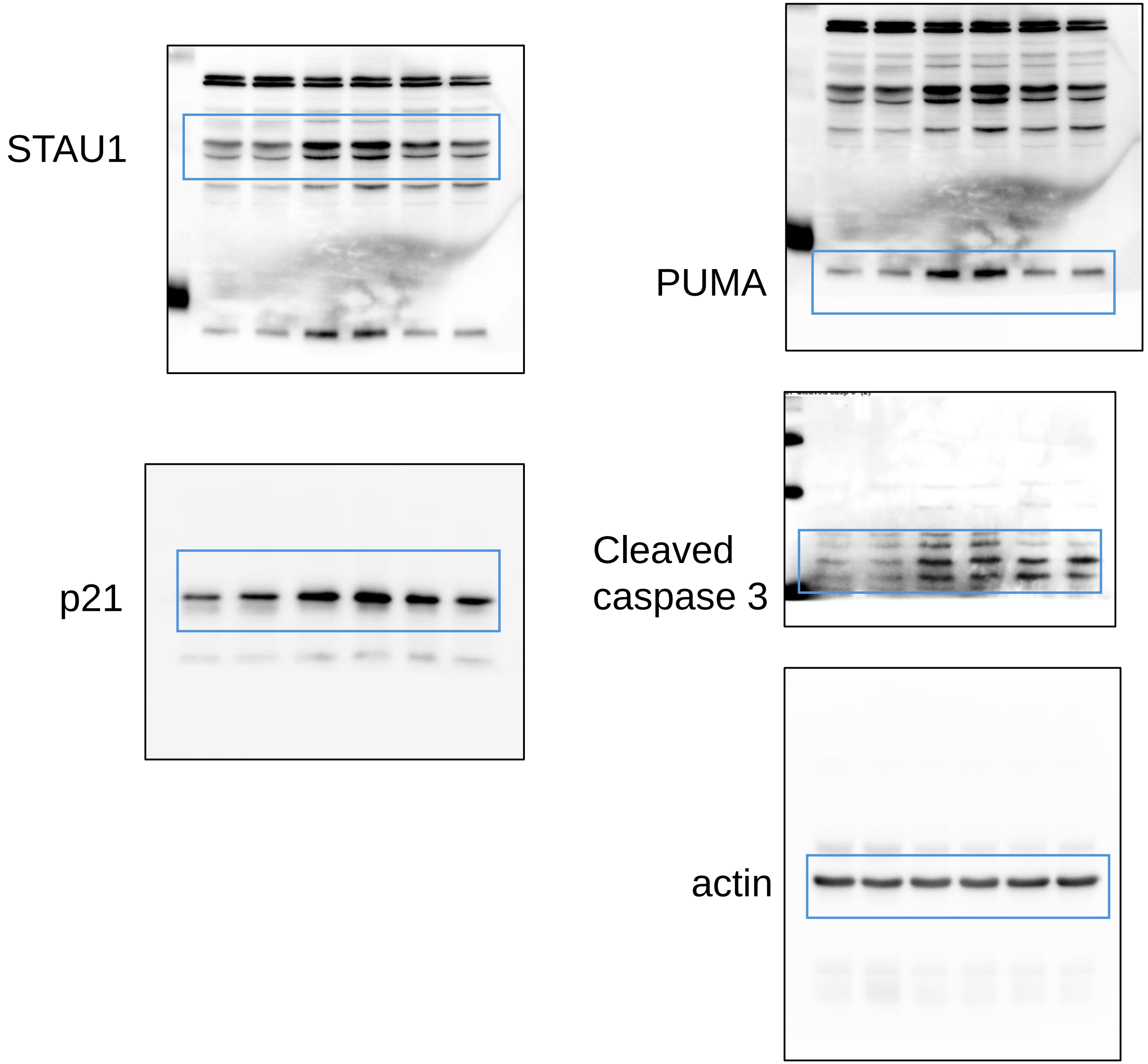

Fig. 6B

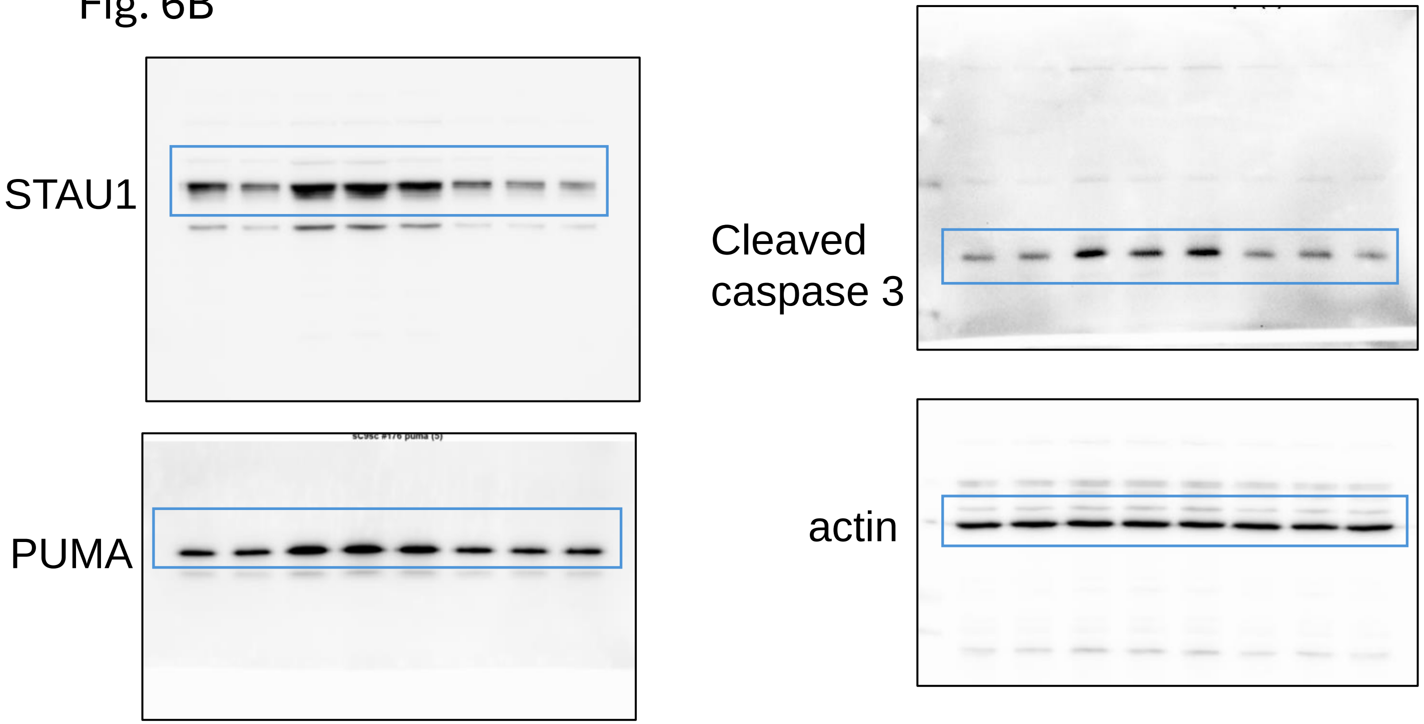

Supplement: Supplementary file 3 — Full length uncropped original Western blots [file 41419_2025_8067_MOESM3_ESM.pdf]
